# Ribosome-associated vesicles: A dynamic subcompartment of the endoplasmic reticulum in secretory cells

**DOI:** 10.1126/sciadv.aay9572

**Published:** 2020-04-01

**Authors:** Stephen D. Carter, Cheri M. Hampton, Robert Langlois, Roberto Melero, Zachary J. Farino, Michael J. Calderon, Wen Li, Callen T. Wallace, Ngoc Han Tran, Robert A. Grassucci, Stephanie E. Siegmund, Joshua Pemberton, Travis J. Morgenstern, Leanna Eisenman, Jenny I. Aguilar, Nili L. Greenberg, Elana S. Levy, Edward Yi, William G. Mitchell, William J. Rice, Christoph Wigge, Jyotsna Pilli, Emily W. George, Despoina Aslanoglou, Maïté Courel, Robin J. Freyberg, Jonathan A. Javitch, Zachary P. Wills, Estela Area-Gomez, Sruti Shiva, Francesca Bartolini, Allen Volchuk, Sandra A. Murray, Meir Aridor, Kenneth N. Fish, Peter Walter, Tamas Balla, Deborah Fass, Sharon G. Wolf, Simon C. Watkins, José María Carazo, Grant J. Jensen, Joachim Frank, Zachary Freyberg

**Affiliations:** 1 Division of Biology and Biological Engineering, California Institute of Technology, Pasadena, CA 91125, USA.; 2 Department of Biochemistry and Molecular Biophysics, Columbia University, New York, NY 10032, USA.; 3 Biocomputing Unit, Centro Nacional de Biotecnología–CSIC, Darwin 3, Campus Universidad Autónoma, 28049 Madrid, Spain.; 4 Department of Psychiatry, University of Pittsburgh, Pittsburgh, PA 15213, USA.; 5 Department of Cell Biology, University of Pittsburgh, Pittsburgh, PA 15213, USA.; 6 HHMI, Department of Biochemistry and Biophysics, University of California, San Francisco, San Francisco, CA 94143, USA.; 7 Department of Cellular, Molecular and Biophysical Studies, Columbia University Medical Center, New York, NY 10032, USA.; 8 Department of Neurology, Columbia University, New York, NY 10032, USA.; 9 Section on Molecular Signal Transduction, Program for Developmental Neuroscience, National Institute of Child Health and Human Development, National Institutes of Health, Bethesda, MD 20892, USA.; 10 Department of Psychiatry, Columbia University, New York, NY 10032, USA.; 11 Division of Molecular Therapeutics, New York State Psychiatric Institute, New York, NY 10032, USA.; 12 Department of Neurobiology, University of Pittsburgh, Pittsburgh, PA 15213, USA.; 13 New York Structural Biology Center, New York, NY 10027, USA.; 14 Department of Pharmacology and Chemical Biology, University of Pittsburgh, Pittsburgh, PA 15261, USA.; 15 CNRS-UMR7622, Institut de Biologie Paris-Seine, Université Pierre & Marie Curie, 75252 Paris, France.; 16 Vascular Medicine Institute, University of Pittsburgh, Pittsburgh, PA 15261, USA.; 17 Center for Metabolism and Mitochondrial Medicine, University of Pittsburgh, Pittsburgh, PA 15261, USA.; 18 Department of Pathology and Cell Biology, Columbia University, New York, NY 10032, USA.; 19 Program in Cell Biology, Hospital for Sick Children, Toronto, Ontario, Canada.; 20 Department of Structural Biology, Weizmann Institute of Science, Rehovot, Israel.; 21 Department of Chemical Research Support, Weizmann Institute of Science, Rehovot, Israel.; 22 HHMI, Division of Biology and Biological Engineering, California Institute of Technology, Pasadena, CA 91125, USA.; 23 Department of Biological Sciences, Columbia University, New York, NY 10027, USA.

## Abstract

The endoplasmic reticulum (ER) is a highly dynamic network of membranes. Here, we combine live-cell microscopy with in situ cryo–electron tomography to directly visualize ER dynamics in several secretory cell types including pancreatic β-cells and neurons under near-native conditions. Using these imaging approaches, we identify a novel, mobile form of ER, ribosome-associated vesicles (RAVs), found primarily in the cell periphery, which is conserved across different cell types and species. We show that RAVs exist as distinct, highly dynamic structures separate from the intact ER reticular architecture that interact with mitochondria via direct intermembrane contacts. These findings describe a new ER subcompartment within cells.

## Teaser

Ribosome-associated vesicles are a novel, dynamic ER-derived organelle.

## INTRODUCTION

The endoplasmic reticulum (ER) constitutes an extensive network of continuous subcompartments distributed throughout the cell (*1*–*3*), comprising the nuclear envelope and includes ribosome-associated rough ER (RER) and ribosome-free smooth ER (*4*–*6*). The advent of higher-resolution imaging modalities has provided new insights into ER organization. Indeed, recent work using super-resolution imaging revealed that ER elements in the cell periphery previously defined as sheets were, in fact, dense tubular structures arranged as ER matrices (*7*, *8*).

Here, we use live-cell super-resolution stimulated emission depletion (STED) microscopy together with highly inclined thin illumination (HiLo) and high-speed three-dimensional (3D) wide-field imaging to visualize ER network dynamics in real time. We have integrated these live-cell imaging approaches with in situ cryo–electron tomography (cryo-ET) and cryo–correlative light and electron microscopy (cryo-CLEM) to visualize the ER and its relationships with other intracellular organelles in near-native states. Notably, the combination of these highly complementary methods has revealed a novel ER-derived compartment that is mobile, vesicular, and associated with mammalian 80*S* cytoplasmic ribosomes. Moreover, with cryo–focused ion beam (cryo-FIB) milling and cryo-ET, we show that these vesicles exist as discrete structures separate from the intact reticular ER architecture. We call these organelles ribosome-associated vesicles (RAVs). Detailed characterization of the RAVs revealed that these structures are conserved across multiple cell types and species using both conventional transmission electron microscopy (TEM) and cryo–electron microscopy (cryo-EM). We also show that RAVs interact with mitochondria via direct membrane contacts, shedding light on the means by which ER and its derivatives communicate with other organelles. Overall, our analyses expand the number of recognized ER subcompartments within cells.

## RESULTS

### Live-cell imaging of dynamic punctate ER

**Fig. 1:**
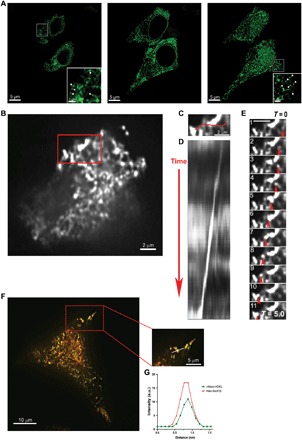
Identification of ER-derived vesicles in secretory cells. (**A**) Live-cell super-resolution STED imaging of insulin-secreting INS-1E cells expressing ER marker mNeon-KDEL. Representative individual optical slices at different planes within the cell including the cell top (left), center (middle), and bottom (right) demonstrate punctate structures primarily in the cell periphery (cell top and bottom), in addition to an extensive reticular distribution throughout the cells. Scale bars, 5 μm. Insets show enlarged images of individual mNeon-KDEL puncta (arrowheads). (**B**) HiLo imaging of INS-1E cells expressing mNeon-KDEL confirms numerous punctate structures (see movies S2 and S3). Scale bar, 2 μm. (**C** to **E**) mNeon-KDEL–labeled puncta demonstrate dynamic movement throughout the cell [including within the boxed region in (B)] using HiLo microscopy. Movement of a mNeon-KDEL punctum is indicated by the following: (C) the horizontal line (in red) to show distance traveled (scale bar, 2 μm), (D) a kymograph of motion across time, and (E) accompanying time-lapse images that show movement at specific time points in the kymograph, as indicated by the red arrows (scale bar, 2 μm). (**F**) Representative HiLo images of INS-1E cells expressing both mNeon-KDEL (in green) and ER membrane marker Halo-Sec61β (in red). Scale bar, 10 μm. Magnified region of interest showing dual-labeled punctate structures within a peripheral process. Scale bar, 5 μm. (**G**) Representative fluorescent line intensity profiles for mNeon-KDEL and Halo-Sec61β channels along the direction of the white line drawn across a puncta showing colocalization of the two ER markers. a.u., arbitrary units.

We visualized the organization of the ER by super-resolution live-cell STED imaging of insulin-secreting pancreatic β-cell–derived INS-1E cells expressing ER marker mNeon-KDEL. Consistent with the ER being an intact network of dynamic membranes, we observed an extensive reticular ER organization throughout the cell (Fig. 1A). Unexpectedly, we also observed apparently punctate mNeon-KDEL–labeled structures predominantly in the cell periphery (Fig. 1A and movie S1). Imaging of multiple optical planes in sequence above and below these structures suggested that the puncta are discrete, isolated structures interspersed with the reticulum (movie S1).

To further characterize the mNeon-KDEL–labeled punctate structures, we applied HiLo microscopy. HiLo microscopy uses a laser directed at a highly inclined angle through the sample, with acquired images numerically processed to reject out-of-focus background signal. This provides high-resolution, diffraction-limited images with a superior signal-to-noise ratio approaching total internal reflection fluorescence (TIRF) imaging, but at greater depths of view (*9*–*11*). These advantages permitted us to visualize punctate structures without being potentially obscured by background fluorescence, particularly when the extensive ER network is densely packed as in the INS-1E cells. Coupled with rapid image acquisition, continuous time-lapse HiLo imaging again revealed a few highly dynamic mNeon-KDEL–labeled putative puncta in the small volume at or near the plasma membrane (Fig. 1, B to E, and movies S2 and S3). Several of these puncta moved over long distances (~5 μm), appearing untethered to the rest of the reticular ER network (Fig. 1, C to E, and movies S2 and S3). Quantitation of puncta size demonstrated a mean diameter of 0.43 ± 0.07 μm (*n* = 33), which was within the range of the punctate structures observed by STED imaging. Labeling cells with other intraluminal ER markers including calreticulin–enhanced yellow fluorescent protein (calreticulin-EYFP) and BiP–green fluorescent protein (BiP-GFP) similarly revealed punctate structures in INS-1E cells (fig. S1, A and B). We additionally examined whether these mNeon-KDEL–labeled puncta colocalized with Sec61β, a membrane-spanning subunit of the ER protein translocation machinery, in cells coexpressing HaloTag Sec61β (Halo-Sec61β) (*12*) labeled with Janelia Fluor 646 HaloTag ligand (JF_646_-HaloTag ligand) (*13*). mNeon-KDEL and Halo-Sec61β showed colocalization of both markers to the punctate structures (Fig. 1, F and G); quantification of the mNeon-KDEL and Halo-Sec61β markers revealed 89% colocalization between the respective signals.

Next, to determine the applicability of our findings to other secretory cell types, we examined primary rat cortical neurons. The thin processes of the dendritic tree in the neuron periphery facilitate identification of small, dynamic structures such as ER-derived puncta. We therefore labeled neuronal ER with mNeon-KDEL and imaged the dendrites via 3D wide-field microscopy, allowing us to capture real-time trafficking with minimal bleaching and maximal sensitivity (fig. S2). We found numerous highly dynamic puncta moving along the length of the dendrites, analogous to the structures observed in INS-1E cells (fig. S2 and movies S4 and S5). Live imaging in neurons and INS-1E cells also revealed that some puncta appeared to stall at times, consistent with the behavior of other mobile intracellular structures such as mitochondria, which stall upon recruitment to sites of local translation in neurons (*14*). Last, these neuronal puncta also colocalized with Halo-Sec61β. In summary, our data suggest that there are ER-derived punctate structures within multiple secretory cell types.

### Cryo-CLEM identification of RAVs

**Fig. 2:**
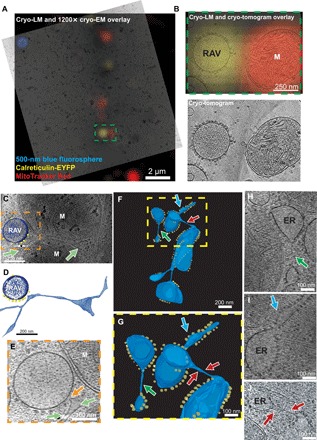
Visualization of ER-derived RAVs by cryo-CLEM and cryo-ET. (**A**) Cryo-CLEM show calreticulin-EYFP localizes to vesicles associated with ribosome-like particles termed RAVs in INS-1E cells. A cryo–tomographic slice was overlaid on an epifluorescence image with calreticulin-EYFP fluorescence in RAVs (in yellow) and MitoTracker Red–labeled mitochondria (in red). Scale bar, 2 μm. (**B**) Enlarged view of the green box in (A) with and without fluorescence overlays show calreticulin-EYFP fluorescence localizes to RAVs with a mitochondrion found alongside (labeled M). Pixels on the detector represent 2.6 Å at the specimen level; images are nonmontaged. Scale bar, 250 nm. (**C**) Cryo–tomographic slice demonstrating a RAV alongside a lamella of conventional ER and mitochondria (indicated by M). Light green arrows indicate cisternae of the ER network. Scale bar, 200 nm. (**D**) Isosurface of the ER-RAV association in (C) highlighting contact between the two structures (ER, light blue; RAV, dark blue; ribosomes, yellow). Scale bar, 200 nm. (**E**) Original cryo-tomographic slice featuring an enlarged view of the area highlighted in the orange box from (C). Light green arrows highlights an ER cisterna sandwiched between the RAV and mitochondrion (M) alongside; the orange arrow points to the site of contact between the ER network and RAV. Scale bar, 100 nm. (**F**) Isosurface showing RAV-like structures attached to ER in mouse embryonic fibroblasts (MEFs) (ER membranes, light blue; ribosomes, yellow). Scale bar, 200 nm. (**G**) Additional, enlarged isosurface view of segmented ER membranes from the yellow box in (F) highlighting attachment sites of RAV-like structures to ER cisternae (indicated by blue, green, and red arrows) including via three-way junctions. Scale bar, 100 nm. (**H** to **J**) Original cryo-tomographic slices featuring views of the thin ER tubules associated with the RAV-like structures highlighted by the respectively colored arrows in (F) and (G). Panels (A to E) in INS-1E cells; Panels (F to J) in MEFs. Scale bars, 100 nm.

Light microscopy remains limited in its ability to provide precise structural details of the ER-derived puncta. These imaging methodologies cannot discern the shape of these structures nor reveal whether the puncta are individual vesicles or clusters of vesicles that move together. Therefore, to further characterize these structures under near-native conditions, we used cryo-CLEM in INS-1E cells expressing the ER marker calreticulin-EYFP. Cryo-light microscopy (cryo-LM) confirmed the presence of calreticulin-EYFP puncta (Fig. 2A). Importantly, the fluorescent calreticulin-EYFP puncta localized to vesicular structures with regularly distributed electron-dense particles conspicuously similar to mammalian ribosomes bound with their cytoplasmic face (Fig. 2B and movie S6). This ribosomal association agrees with colocalization of Sec61β to the puncta according to our live-cell imaging. Using cryo-EM, we also found that the vesicular organelles were nearly circular (eccentricity, 0.19 ± 0.02) in projection. Consequently, we designated these structures RAVs.

Since RAVs share a similar vesicular morphology with dense core secretory granules (DCSGs), which are abundant in secretory cells (*15*), we set out to test whether RAVs are a novel subset of these vesicles. Specifically, we asked whether a canonical DCSG marker, chromogranin A (CgA) tagged with enhanced green fluorescent protein (EGFP) (CgA-GFP) (*16*), colocalized with RAVs. Live imaging of CgA-GFP in INS-1E cells revealed a punctate expression pattern throughout the cytoplasm (fig. S1C), consistent with the established intracellular distribution of this specific secretory granule marker (*17*). We then determined whether fluorescent CgA-GFP puncta localized to the RAV lumen using cryo-CLEM (fig. S3, A to C). CgA-GFP–labeled puncta colocalized with DCSGs, granules known to contain CgA (*16*, *18*), but not to RAVs (movie S7). We also found that a fraction of DCSGs, including those labeled with CgA-GFP, exhibited intraluminal crystalline structures, in line with earlier EM studies (fig. S3, B and C) (*19*, *20*). The power spectrum of the 2D image of the crystals exhibited a hexagonal lattice (fig. S3, D to F). This is consistent with a 2D projection of a crystalline arrangement of insulin with rhombohedral symmetry, which is the previously described 3D arrangement of insulin crystals in vitro (*21*, *22*). Notably, we did not observe these crystals in the RAVs. Since insulin crystals localize to mature DCSGs (*23*–*25*), the absence of these crystals in RAVs suggests that this compartment is likely distinct from DCSGs.

### Interactions between RAVs and ER

Cryo-ET imaging showed that RAVs and ER interact closely via direct contacts between the respective membranes of both structures (Fig. 2, C to E). We also observed membrane contacts providing potential continuity between RAV-like structures and the ER network via thin membrane tubules (Fig. 2, F to J). In some instances, RAV-like structures remained attached to the main ER network as part of three-way junctions (Fig. 2, F and G), providing further evidence that RAVs are related to the main ER network.

### Subtomogram averaging of RAV-associated ribosomes

**Fig. 3:**
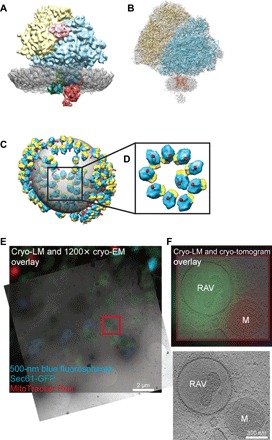
Subtomogram average of RAV-bound ribosomal complex. (**A**) Average of 1362 manually selected subvolumes of RAV-bound electron-dense particles. Reference-free alignment reveals that these particles strongly resemble 80*S* mammalian ribosomes. Putative density assignments: yellow, 40*S* subunit; light blue, 60*S* subunit; purple, tRNA-eEF1a-GTP ternary complex; gray, averaged portion of RAV membrane associated with the bound ribosome; dark blue, translocon; green, TRAP; red, OST. (**B**) Subtomogram average fitted over a color-coded, high-resolution atomic model of the mammalian ribosome-Sec61 complex (EMD 2644) (*31*), revealing similarity between the structures. Yellow, 40*S* subunit; blue, 60*S* subunit; red, Sec61. Additional densities potentially representing OST and TRAP are also evident (in gray). (**C**) Illustration of subtomogram averaged RAV-bound ribosomes mapped onto their original positions on a RAV. Bound particles show an ordered spiral arrangement indicative of polysomes. This illustration was created by mapping a bound-ribosome subtomogram average into the original locations of the subtomograms containing putative ribosomes. (**D**) Enlarged view of the spiral polysome arrangement. Orientations of ribosomal exit tunnels (in red) are depicted relative to the sphere in (C) and (D). (**E**) GFP-Sec61β localizes to RAVs by cryo-CLEM. A cryo-tomographic slice correlated with an epifluorescence image demonstrating GFP-Sec61β fluorescence in RAVs (in green) and MitoTracker Red–labeled mitochondrion (in red); 500-nm fiducial blue fluorospheres (in blue) are also evident. Scale bar, 2 μm. (**F**) Enlarged cryo-tomogram views of the red boxed area in (E) with and without fluorescence overlays. GFP-Sec61β fluorescence localizes to the population of RAVs. Pixels on the detector represent 2.6 Å at the specimen level; images are nonmontaged. Scale bar, 300 nm.

We used cryo-ET to confirm the identity of the putative 80*S* ribosomes bound to RAV membranes. The diameter of the electron-dense particles associated with the membranes of the RAVs, 320 Å, fits with the dimensions of mammalian ribosomes (*26*, *27*). To determine the identity of the particles, we extracted subtomograms containing putative membrane-bound ribosomes. To avoid reference bias, we used reference-free alignment and averaging of subtomograms (*28*). The average of 1230 subtomograms matched well with the structure of a mammalian 80*S* ribosome (fig. S4A and movie S8). Both the 40*S* and 60*S* ribosomal subunits were present, as well as additional putative components of the translational machinery, including the amino acid–transfer RNA (tRNA)–eukaryotic translation elongation factor 1a (eEF1a)–guanosine 5′-triphosphate (GTP) ternary complex. Within the bound ribosomal complex, densities likely to be oligosaccharyltransferase (OST) and translocon-associated protein (TRAP) complexes were associated with a putative translocon embedded within the RAV membrane (Fig. 3A) based on earlier studies of these complexes (*29*, *30*). We determined the resolution of the average RAV membrane–bound complex to be 15 Å by Fourier shell correlation (FSC) comparison of our subtomogram average with the subtomogram average of the mammalian ribosome (fig. S4B) (*26*). Our map of the averaged RAV-bound 80*S* ribosomal complex was also fitted to a high-resolution atomic model of the mammalian ribosome-Sec61 complex (*31*), revealing strong similarity between the structures (Fig. 3B). We conclude that the membrane-bound particles are indeed ribosomes, justifying the designation of RAVs.

### Arrangement of ribosomes on RAVs

We obtained a 3D contour of the RAV membrane by manual segmentation of the cryo-tomogram, onto which copies of the averaged ribosome were mapped back at the locations and orientations where the individual 80*S* ribosomes were found (Fig. 3C). Notably, RAV-bound ribosomes showed a spiral-like arrangement on the spherical membrane surface (Fig. 3D and movie S9). This spiral rosette–like arrangement had been previously described for mammalian polysomes in both conventional EM and cryo-ET studies (*32*, *33*) and by atomic force microscopy (*34*). These ribosomes had an average nearest-neighbor distance (NND) of 32 ± 5 nm, similar to previously reported NND values for human polysomes (*32*). Mapping of individual unbound ribosomes in the cytoplasm and RAV-bound ribosomes from cryo-tomograms revealed chains of equally spaced ribosomes approaching the RAV membranes from the cytoplasm (fig. S5). These linear strings of unbound ribosomes in the cytoplasm were continuous with the arrangement of RAV-bound ribosomes (fig. S5 and movie S10). Moreover, the exit tunnels of >90% of all ribosomes on RAVs projected directly into the RAV lumen, including the ribosomes assembled as polysomes (Fig. 3, C and D). Ribosomes in these polysomal assemblies have long been associated with active translation (*34*–*36*). Therefore, the combined evidence of (i) ribosomal orientations, (ii) polysomal assembly, and (iii) presence of the putative ternary complex suggests that the RAV-bound 80*S* ribosomal complex is likely translationally active. In addition, we used cryo-CLEM to determine whether the Sec61 translocon was present in RAV-bound ribosomal complexes. Using GFP-tagged Sec61β (GFP-Sec61β) expressed in INS-1E cells, we found that GFP fluorescent signal localized to RAVs. Upon quantification, we found that 100% of GFP-Sec61β signal correlated to RAVs, enabling their identification via cryo-CLEM (*n* = 13 RAVs in two separately imaged cells) (Fig. 3, E and F). These data confirm the presence of the Sec61 translocon within the RAV-bound ribosomes.

We identified a subset of RAVs with internal membranes (Fig. 3F). The classical ER reticulum consists of interconnected fenestrated membrane sheets that are linked to one another by tubules (*37*). We posit that dynamic changes within this fenestrated interconnected ER give rise to intraluminal vesicles that are occasionally visualized as internal membranes. In addition, the consistent presence of cytoplasmic ribosomes associated with RAV outer membranes argues against RAVs representing multilamellar bodies or autophagosomes. Comparison of RAVs with classical double-membrane autophagosomes demonstrates the fundamental morphological differences between these two classes of structures (fig. S6).

### Intracellular RAV distribution

**Fig. 4:**
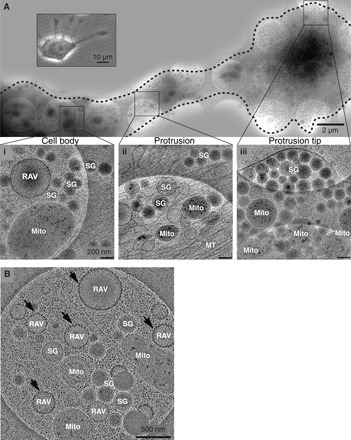
Distribution of RAVs in the cell periphery. INS-1E cells were grown directly on EM grids. (**A**) Overview cryo-EM image of a thin protrusion extending from the periphery of the cell body and containing accumulations of secretory granules, ribosomes, and cytoskeleton. Scale bar, 2 μm. Inset shows a representative phase-contrast image of an INS-1E cell grown on an EM grid; this image is from a different cell than the one imaged in the remainder of the figure. Scale bar, 10 μm. (**i**) At the junction between the cell body and the protrusion, there are accumulations of mitochondria, small dense-core secretory granules, and RAVs. (**ii**) Extensive microtubule tracks run lengthwise along the protrusion and appear to transport secretory granules and mitochondria toward (**iii**) the expanded tip of the protrusion. These tips are packed with mature dense-core secretory granules and mitochondria and are absent of RAVs. Scale bars, 200 nm. (Note that white circles correspond to 2-μm holes of the QUANTIFOIL holey carbon film onto which the cells are attached). (**B**) An additional view of the same junctional region between the cell body and protrusion highlighted in (i) featuring an abundance of RAVs (indicated by arrows) and free, cytoplasmic ribosomes. Scale bar, 500 nm. Pixels on the detector represent 2.6 Å at the specimen level; images are montaged in the overview with nonmontaged images in the remaining panels. Images are representative of *n* > 3 independent experiments.

We used in situ cryo-EM to characterize the intracellular distribution of RAVs in relation to components of the secretory machinery, including secretory vesicles within INS-1E cells. Focusing on the thinner, cryo-EM–accessible region parts of the cell, we found that RAVs were consistently concentrated at the junction between the cell body and the base of cell protrusions in proximity to numerous mitochondria (Fig. 4, Ai and B). In the same region, we also found secretory granules that were surrounded by large numbers of unbound ribosomes (Fig. 4Ai). Distal to the cell junction, within the cell protrusion, we discerned microtubule tracks running lengthwise along these extensions that appeared to transport secretory granules and mitochondria toward the expanded tip of the protrusion (Fig. 4Aii). The tips of these protrusions were densely packed with secretory granules (Fig. 4Aiii). We also quantified the intracellular distribution of these organelles relative to secretory granules and ribosomes using stereological approaches (*38*–*40*). Secretory granules were significantly more concentrated in the cell protrusions and their tips compared to the cell body, whereas free ribosomes were significantly enriched in the cell body compared with the protrusion *(P <* 0.0001; fig. S7, A and B). By comparison, the highest RAV density was in the cell body *(P =* 0.0005; fig. S7C). Consistent with this observation, RAVs constituted 15.7% of the total vesicle pool in the regions of the cell body we imaged but <1% of the total vesicle pool in the cell protrusion tips; vesicles were defined as approximately spherical, membrane-bound objects discontinuous from other intracellular membranes.

### Intracellular visualization of ER and Golgi apparatus by cryo-FIB milling

**Fig. 5:**
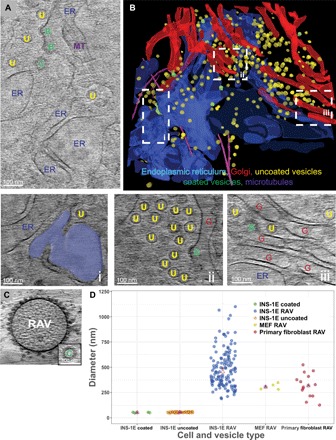
Cryo-tomography of cryo-FIB milled INS-1E cells reveals the intact ER and Golgi apparatus secretory complex. (**A**) Cryo–tomographic slice of a cryo-FIB milled region in the cell center exhibiting flattened ER cisternae (labeled ER) and coated membrane sites of vesicle budding (labeled B). Alongside these apparent exit sites are both coated and uncoated vesicles (labeled C and U, respectively) and microtubules crisscrossing the field (labeled MT). Scale bar, 100 nm. (**B**) Colorized 3D segmentation of the same field demonstrating interconnected ER (in blue) and Golgi apparatus (in red). Interspersed throughout are coated vesicles (in green), uncoated vesicles (in yellow), and microtubules (in magenta). (**i** to **iii**) Enlarged panels highlighting key structural features: (i) segmented ER cisterna within the larger ER network with an intact uncoated vesicle alongside; (ii) Golgi cisterna including a site of coated membrane budding with numerous uncoated vesicles nearby; (iii) additional view of the Golgi membrane network featuring a site of coated membrane budding and uncoated vesicles. Scale bars, 100 nm. (**C**) Cryo–tomographic slices comparing the appearance and dimensions of a RAV (~200-nm diameter) with a conventional coat protein complex (COP)–coated vesicle (~50-nm diameter). Scale bar, 100 nm (corresponds both to the main panel and the inset). (**D**) Scatter plot of the distribution of diameters of vesicular structures including RAVS across several cell types and species [rat INS-1E–coated vesicles, INS-1E uncoated vesicles, INS-1E RAVs, mouse embryonic fibroblast (MEF) RAVs, and human primary fibroblast RAVs]. Asterisks represent the median diameter, and triangles represent the mean diameter of the respective vesicles.

To visualize the ER network in thicker, central areas of the cell less accessible to cryo-EM, we used successive cryo-FIB milling on both sides of the cell to generate thin, 100- to 300-nm-thick lamellae (fig. S8). We found an extensive intact ER including apparent ER exit sites on selected cisternae that appeared to be releasing coated vesicles (Fig. 5A). These sites were surrounded by both coated and uncoated vesicles, providing a snapshot of vesicular transit within the secretory pathway (Fig. 5, A and B) consistent with a previous snapshot of vesicular transport in situ (*41*). Our 3D cryo-tomograms acquired within the cryo-FIB milled lamellae revealed that the ER and Golgi apparatus appear interconnected through numerous close membrane associations (Fig. 5B and movie S11). The fact that these two organelles exist in conformations similar to those observed earlier via room temperature tomography of freeze-substituted and resin-embedded cells (*42*) suggests that their structures and relative spatial relationships are not disrupted in our cryo-ET cell preparations. Ultimately, the presence of an intact ER network in our cells further argues against RAVs being generated in the experiment as a consequence of structural disruptions to the existing secretory machinery.

Cryo-FIB milling also revealed a considerable population of small coated and uncoated vesicles in the cell center [Fig. 5, A and B (ii and iii)]. In contrast, we found relatively few RAVs in the cell center in the vicinity of the ER and Golgi apparatus; RAVs were found predominantly in the cell periphery. Comparison of ER-derived coated vesicles with RAVs further emphasized the respective differences between these vesicular structures (Fig. 5C). Small coated vesicles in our cryo-tomograms had a 51.7 ± 3.7–nm diameter, consistent in both size and appearance with earlier reports (*41*). Moreover, whereas the size of both coated and uncoated vesicles was highly uniform, RAVs in INS-1E cells were larger and more heterogeneous (mean diameter, 496 ± 204 nm; Fig. 5D).

### Visualization of RAVs in other cell types

Although our initial characterization of RAVs was in rat pancreatic β-cell–derived INS-1E cells, we also found RAVs in cells from several other tissues and species. For example, RAVs were observed in primary human fibroblasts (fig. S9, A and B), mouse embryonic fibroblasts (MEFs) (fig. S9C), and human BE(2)-M17 cells, a dopamine-secreting neuron-derived cell line (fig. S9D) (*43*). As in INS-1E cells, RAVs in other cell types were spherical, although with smaller diameters (309 ± 32 nm in MEFs and 321 ± 119 nm in primary fibroblasts; Fig. 5D). Consistent with our earlier observations, RAVs in these additional cell types were primarily found in the cell periphery near microtubule tracks and, in some instances, near intact ER reticulum (fig. S9, C and D). The presence of RAVs in nonimmortalized primary cells and in untreated, untransfected immortalized cells suggests that these organelles are not an artifact of cell immortalization or other forms of extrinsic cell manipulations.

We independently confirmed our cryo-EM and cryo-ET findings via cryo–scanning transmission electron tomography (CSTET), a form of cryo-ET that permits us to acquire 3D views of intact, unfixed cells through much thicker volumes as compared to cryo-TEM (*44*). Using CSTET, we also observed RAVs mainly in the cell periphery in two additional untreated, unfixed cell types grown under standard culture conditions: WI-38 human embryonic lung fibroblast cells (fig. S10, A and B) and human dermal microvascular endothelial cells (HDMECs) (fig. S10, C to E, and movie S12). Together, our observations suggest that RAVs are conserved across different species and cell types and can be observed through a wide variety of imaging modalities.

### RAV-mitochondrial interactions

**Fig. 6:**
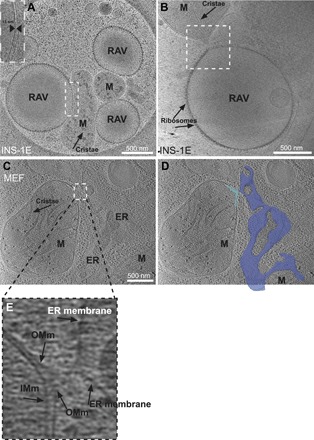
RAV- and ER-mitochondrial interactions imaged by cryo-EM and cryo-ET. (**A**) Cryo-EM image of interactions between RAVs and nearby mitochondria (labeled M) in INS-1E cells. Inset shows enlarged view of the interface between RAV and mitochondrial membranes. Note the absence of ribosomes at the tight interface (13-nm width). Scale bar, 500 nm. (**B**) Site of RAV-mitochondrion interaction in which RAV and mitochondrial membranes extend, acquiring an “hourglass-like” morphology at the single point of contact (within the boxed area). Scale bar, 500 nm. (**C**) Cryo–tomographic slice of a MEF, revealing an ER-mitochondrial contact site. Scale bar, 500 nm. (**D**) Segmentation of the ER-mitochondrial association in MEFs highlighting a similar hourglass-like point of membrane contact (ER, dark blue; mitochondrion, light blue) point of contact. (**E**) Enlarged view of the ER-mitochondrial contact in (C) showing the extension and deformation of both the OMm and ER membrane. Both the inner mitochondrial membrane (IMm) and mitochondrial cristae remain unchanged.

Our cryo-EM data showed that RAVs made direct contacts with mitochondria. At times, the membranes of the two organelles were observed in close apposition (13-nm distance), such that RAV-bound ribosomes were excluded at the tight membrane interface (Fig. 6A). In other cases, RAV and mitochondrial membranes were deformed to meet in an hourglass-like contact, as observed in INS-1E cells (Fig. 6B). This hourglass-like membrane deformation strongly resembled the contact sites we observed between ER and mitochondrial membranes (i.e., mitochondria-associated membranes, or MAMs) in MEFs (Fig. 6, C and D). In both cases, although the outer mitochondrial membrane (OMm) and RAV or ER membranes were highly distorted at the point of contact, reaching toward each other at sharp points, the inner mitochondrial membrane and mitochondrial cristae remained unaltered (Fig. 6E). Quantitation of RAV-mitochondrial contacts across our cryo-ET datasets revealed that RAV-mitochondrial interactions were present in 25% of the cryo-tomograms, which displayed both RAVs and mitochondria in the same field (in *n* = 26 of 104 cryo-tomograms surveyed).

### Analysis of cell stress and cytotoxicity

Our data show RAVs to be a highly conserved compartment found across diverse cell types, species, and culture conditions. Moreover, we regularly observe these structures at the cell periphery, particularly in our cryo-ET and cryo-CLEM experiments targeting ER markers. Nevertheless, we explored the possibility that RAVs arise primarily due to cytotoxicity and/or cell stress. We first examined whether growth of mammalian cells on gold cryo-EM grids under the conditions we used to image RAVs could lead to gross cellular morphological abnormalities or cell death. Both confocal and differential interference contrast (DIC) imaging of INS-1E cells grown on fibronectin-coated gold cryo-EM grids under the conditions used for our studies demonstrated adherent cells with well-defined morphology and extensive cell spreading (fig. S11, A to D, and movie S13); these observations were identical to images of healthy, proliferating INS-1E cells grown conventionally in tissue culture dishes (*45*, *46*). Moreover, time-lapse DIC movies of grid-grown cells demonstrated the cells to be highly dynamic with extensive cell movement and cortical activity/remodeling over time (movie S13). We also tested for potential cytotoxicity and cell death in response to culturing cells on cryo-EM grids using a fluorescence-based cell viability assay. We found that >99% of INS-1E cells grown on cryo-EM grids for >48 hours were labeled with a green fluorescent calcein–AM (acetoxymethyl) viability dye, suggesting that the cells on the grids were viable. In contrast, cell labeling with red fluorescent ethidium homodimer-1, an indicator of cytotoxicity and cell death, was virtually absent in all fields assayed (fig. S11, A to D). This indicated that growth on the cryo-EM grids for extended periods did not induce notable cytotoxicity leading to cell death.

We determined whether growing cells on cryo-EM grids affected cell energy metabolism, a sensitive indicator of potential intracellular derangements. We examined mitochondrial respiration via oxygen consumption rates (OCRs) using Seahorse extracellular flux analysis in INS-1E cells grown directly on cryo-EM grids versus cells conventionally cultured in assay plates. Cryo-EM grid–grown cells exhibited no significant differences in basal respiration compared with the control dish–grown cells (*P* > 0.05; fig. S11E). Furthermore, grid-grown cells responded in a similar manner as dish-grown cells to mitochondrial stress tests examining changes in OCR in response to sequential inhibition of different components of mitochondrial respiration (fig. S11E). In parallel, we also examined whether our culture conditions induced ER stress in INS-1 cells, the parental cell line for INS-1E cells (*45*). We assayed for several ER stress markers including phospho-PKR-like ER kinase (p-PERK) and phosphorylated eukaryotic translation initiation factor 2α (p-eIF2α) by immunoblot; we also measured levels of spliced X-box binding protein 1 (XBP1), an indicator of inositol-requiring enzyme 1α (IREα) activation, by reverse transcription polymerase chain reaction (RT-PCR). There was no evidence that these stress markers were induced in cells grown under our culture conditions (fig. S11, F to H). In contrast, treatment with thapsigargin or tunicamycin, potent inducers of ER stress, caused emergence of the above stress markers within 1 hour of treatment and the appearance of ER stress–induced proapoptotic transcription factor C/EBP homologous transcription factor (CHOP) with longer treatments (fig. S11, F to H). Furthermore, while INS-1E cells are commonly grown in the presence of small amounts of β-mercaptoethanol to generate a reducing environment that prevents accumulation of toxic oxygen radicals (*45*), our data show that this does not generate ER stress in the cells. It is highly unlikely that β-mercaptoethanol induces RAV formation since we also observed RAVs in a multitude of cell types not grown in β-mercaptoethanol including primary human fibroblasts, MEFs, BE(2)-M17 cells, WI-38 cells, or HDMECs. By a similar rationale, it is unlikely that RAVs are a consequence of cell transfection since most of cells that exhibited RAVs were untransfected. In summary, none of these tests indicated that RAVs were products of abnormal cell culture conditions, growth on EM grids, or cell stress/toxicity.

### Conventional TEM evidence of RAVs

We observed possible examples of RAVs by conventional TEM with the caveat that at least a fraction of these ribosome-associated structures may represent parts of extended ER tubules viewed in 2D cross section. Nevertheless, we observed putative RAVs within a peripheral cell extension of a WI-38 fibroblast (fig. S12) and in the periphery of human adrenocortical SW-13 cells (fig. S13), where we found multiple examples of RAV-mitochondrial interactions (fig. S13, B to E), consistent with our cryo-ET data. Importantly, we observed putative RAVs in developing primary hippocampal neurons in dendrites along microtubule tracks in proximity to membrane protrusions (fig. S14A) and within these protrusions (fig. S14A, iii). Examination of the perinuclear region of the neuronal cell body revealed intact, extensive secretory machinery including RER and numerous Golgi stacks (fig. S14B), suggesting that the putative RAVs were not simply the result of ER vesiculation in primary neurons.

### Evidence of RAVs in prior studies

The conservation of RAVs across diverse cell types and species as well as via different imaging approaches raises the question: If these organelles are widespread, at least in secretory cells, then why have they not been described in the literature thus far? We found numerous examples of RAVs in conventional TEM micrographs as well as in cryo-EM and cryo-ET data within the published literature (fig. S15). However, in earlier studies, the presence of RAVs was either not explicitly commented upon or analyzed. Alternatively, RAVs were identified as RER, despite their vesicular, nonreticular morphology. For example, Medalia and colleagues (*47*) demonstrated structures entirely consistent with RAVs in the peripheral region of a *Dictyostelium discoideum* cell, simply terming these structures “ER.” Both conventional TEM (fig. S15A) and cryo-ET (fig. S15, B to D) provided 2D and 3D evidence of RAVs, respectively, in *Dictyostelium discoideum* (note the similarity between the RAV from *Dictyostelium* in fig. S15D and our 3D reconstructions of RAVs in fig. S5). Importantly, the presence of RAVs in *Dictyostelium*, a cell type that was simply placed on the grids shortly before cryo-preservation, further supports the idea that these structures are physiologically relevant rather than an artifact of culture conditions. We similarly found several other examples of RAVs in earlier cryo-ET studies of MEFs (fig. S15E), as well as putative RAVs in TEM imaging of high-pressure frozen, freeze-substituted cells including human HepG2 human hepatoblastoma cells (fig. S15F) and African green monkey Vero kidney epithelial cells (fig. S15G).

## DISCUSSION

We characterized RAVs through a combination of light and EM imaging modalities across several scales of resolution. At the light level, our live-cell STED, HiLo, and wide-field imaging data in both INS-1E cells and primary neurons reveal the presence of dynamic ER-derived puncta, especially in the cell periphery. Colocalization of these apparently punctate structures with both luminal and membrane ER markers strongly suggests that these structures represent a subset of the ER. While it may be challenging to definitively distinguish some of the punctate structures from components of ER tubules by light microscopy approaches, especially within individual optical planes, at the electron microscopy level, cryo-ET definitively revealed the presence of isolated spherical vesicles decorated by actively translating ribosomes that we termed RAVs.

Cryo-ET– and cryo-FIB milled data revealed that RAVs coexist with an intact ER network within cells. These findings, together with analyses showing the absence of major cell stress in our samples, suggested that the presence of RAVs was not a direct consequence of stress-induced disruption of the ER. Nevertheless, we cannot rule out the possibility that constitutive secretory activity and its effects on ER structure and function may contribute to RAV formation or numbers. Many components of the protein folding and quality control machinery are endogenously up-regulated to maintain ER function in response to the high levels of protein translation and secretion found in cells specialized for secretion (*48*, *49*). Therefore, while these physiological states may catalyze emergence of RAVs from the ER, on a functional level, RAVs may represent a new mechanism by which secretory cells keep up rapidly with the intensive demands of protein synthesis, especially in a highly localized manner. Furthermore, since protein synthesis demands are often cell type specific (*50*), this may explain the relative heterogeneity of RAVs in terms of size, shape, and/or numbers across different cell types and species that we observed both in the earlier literature and in our own data. Membrane-bound organelles such as the ER and mitochondria are highly dynamic, capable of changing morphology, size, and numbers in response to the specific protein synthesis requirements of different cell types or cell states (e.g., periods of heightened cellular activity) (*51*–*53*).

The mechanisms by which RAVs emerge from the ER are unknown. We suggest that changes in local membrane curvature along the ER tubular membrane network give rise to RAVs. Previous studies proposed that the cooperative interplay of ER membranes with curvature-stabilizing proteins including reticulons, lunapark protein, and the membrane-fusing guanosine triphosphatase (GTPase) atlastin is important in the shaping and remodeling of the tubular ER membrane network (*54*–*56*). Consistent with this, dominant-negative GTPase-defective atlastin mutants produced long, unbranched ER membrane tubules along with ER-derived vesicles (*56*). The presence of these RAV-like structures along ER tubular junctions, sites that favor localization of curvature-stabilizing proteins such as lunapark (*55*), also suggests involvement of the ER-shaping machinery in RAV biogenesis. It is therefore possible that changes in the relative stoichiometry, function, and/or membrane distribution of the ER-shaping machinery lead to alterations in ER membrane curvature that are energetically favorable for RAV formation.

In developing neurons, our visualization of RAVs localized to dendrites may be relevant to the emerging understanding of local translation. Local translation is increasingly recognized as an essential contributor to activity-dependent synaptic plasticity and neuron remodeling (*57*, *58*). Despite thousands of mRNAs trafficked to dendrites presumably for site-specific translation, the machinery for local translation of the protein products remains poorly defined (*59*). RAVs may therefore represent a new mechanism for local translation, facilitating functional coupling between cell activity and protein synthesis at defined sites in the cell periphery. RAV-driven local translation in dendrites would require less time and energy than the traffic of mRNAs or translated products from conventional ER in the cell body.

The relationship of RAVs with mitochondria may provide further insights into RAV function. We show that the membranes of both RAVs and mitochondria can link to each other at sites of contact, mirroring ER-mitochondrial MAMs (*60*, *61*). Similar to MAMs, it is therefore possible that RAV-mitochondrial contacts may play critical roles in functions including membrane biogenesis and Ca^2+^ signaling (*62*, *63*). Consistent with our findings, earlier work reported contacts between mobile ER-derived structures and mitochondria, implicating these interactions in Ca^2+^ exchange between the two organelles (*64*). Similarly, studies in dendrites of hippocampal neurons described a dynamic vesicular ER subcompartment that may serve as a highly mobile Ca^2+^ storage site (*65*). Collectively, these findings suggest that mobile ER-derived compartments, including RAVs, may be responsible for spatial and temporal regulation of local translation, Ca^2+^ signaling, and mitochondrial function—processes that fundamentally contribute to activity-dependent plasticity (*64*, *66*). This work raises a number of important questions, including whether the ribosomes associated with RAVs translate a unique subset of proteins and whether these structures bear specific targeting machinery different from conventional ER for this translation. Future work will also define the mechanisms responsible for mRNA trafficking to specific sites of RAV-driven local translation as well for the membrane deformations producing RAV-mitochondrial membrane contact sites.

## MATERIALS AND METHODS

### Reagents

#### Chemicals

All chemicals used in the present study were purchased from Sigma-Aldrich (St. Louis, MO). MitoTracker Red was purchased from Thermo Fisher Scientific (Pittsburgh, PA). JF_646_-HaloTag ligand was provided by L. Lavis (Janelia Research Campus, Howard Hughes Medical Institute, Ashburn, VA) and was derived from previously reported parent compounds (*13*).

#### DNA constructs

mNeon-KDEL consists of a leader sequence from mouse immunoglobulin κ light chain 5′ to mNeonGreen, followed by a KDEL ER retrieval sequence 3′ to the fluorophore. BiP-GFP contains at the 5′ end a hamster BiP signal sequence, followed by the hamster BiP complementary DNA (cDNA) sequence fused to superfolder GFP (sfGFP), followed by a KDEL ER retrieval sequence at the 3′ end (i.e., 5′-hamster signal sequence-hamster BiP cDNA-sfGFP-KDEL-3′; gift of E. Snapp, Janelia Research Campus, Ashburn, VA). Calreticulin-EYFP consists of the calreticulin signal sequence immediately 5′ to the EYFP gene, followed by a KDEL ER retrieval sequence at the 3′ end (pEYFP-ER; Clontech Laboratories Inc., Mountain View, CA). Sec61-based constructs were described earlier including Halo-Sec61β (gift of J. Bewersdorf, Yale University) (*12*) and GFP-Sec61β (*67*, *68*). Phosphatidylinositol synthase (PIS)–GFP consists of human PIS fused to EGFP at the 3′ end and was described in detail previously (*69*). CgA-GFP consists of full-length human CgA tagged with EGFP at its 3′ end as described previously (*16*).

### Animal husbandry

Animals were housed and handled in accordance with all appropriate NIH guidelines through the University of Pittsburgh Institutional Animal Care and Use Committee (IACUC). We abided by all appropriate animal care guidelines including ARRIVE (Animal Research: Reporting of In Vivo Experiments) guidelines for reporting animal research (*70*, *71*). Rats were housed in cages with a 12:12 light:dark cycle and had access to food and water ad libitum at all times. Studies were performed in accordance with a University of Pittsburgh IACUC–approved protocol (#19024465), and all efforts were made to ameliorate animal suffering.

### Tissue culture

#### Cell line culture

Rat INS-1E cells (gift of P. Maechler, Université de Genève) were cultured in Roswell Park Memorial Institute (RPMI) 1640 medium (Life Technologies, Grand Island, NY) supplemented with 2 mM l-glutamine, 5% heat-inactivated fetal bovine serum, 10 mM Hepes, penicillin (100 U/ml), streptomycin (100 μg/ml), 1 mM sodium pyruvate, and 50 μM β-mercaptoethanol as described earlier (*45*) (termed complete serum-supplemented RPMI 1640 medium). All INS-1E cells used here were found negative for mycoplasma contamination. Primary human fibroblasts were cultured in Dulbecco’s modified Eagle’s medium (DMEM) (Life Technologies) supplemented with 15% fetal bovine serum, 1 mM sodium pyruvate, 1% Minimum Essential Medium (MEM) vitamin solution (Thermo Fisher Scientific), and 1% antibiotic-antimycotic solution (Thermo Fisher Scientific). MEF cells were maintained in DMEM supplemented with 10% fetal bovine serum, penicillin (10 U/ml), and streptomycin (10 μg/ml) (Life Technologies). Human neuroblastoma BE(2)-M17 cells (CRL-2267, American Type Culture Collection, Manassas, VA) were cultured in Opti-MEM medium (Life Technologies) supplemented with 10% fetal bovine serum, 2 mM glutamine, penicillin (100 U/ml), and streptomycin (100 μg/ml). Human adrenocortical SW-13 cells were cultured in L-15 medium supplemented with 10% fetal calf serum, penicillin (0.06 mg/ml), streptomycin (100 μg/ml), and Fungizone (10 μg/ml), buffered with l-arginine at pH 7.4 (Invitrogen, Carlsbad, CA). Embryonic lung fibroblast WI-38 cells were cultured in minimal essential medium (Gibco, Gaithersburg, MD) supplemented with 15% fetal calf serum, l-glutamine, and penicillin/streptomycin. HDMECs (gift of R. Alon, Weizmann Institute) were grown in endothelial cell growth medium (PromoCell, Heidelberg, Germany). All cell lines were maintained in a humidified 37°C incubator with 5% CO_2_.

#### Primary neuron culture

Dissociated rat cortical interneurons were isolated and cultured as described previously (*72*). Briefly, cortical neurons were prepared from embryonic day 18 (E18) Long-Evans rats (Charles River Laboratories, Wilmington, MA). Cortical neurons were plated onto 35-mm-diameter circular glass-bottom dishes (MatTek, Ashland, MA) coated with poly-d-lysine (20 μg/ml) and laminin (3.4 μg/ml) at a density of 3.25 × 10^5^ cells/ml (total volume, 2 ml). The neurons were maintained in neurobasal medium supplemented with B27, penicillin/streptomycin (100 U/ml and 100 mg/ml, respectively), and 2 mM glutamine. One-fifth of the medium in each dish was replaced every 4 days. Dissociated rat hippocampal neurons were cultured as described previously (*73*–*75*). Briefly, hippocampal neurons were obtained from E18 Sprague-Dawley rats and plated onto poly-l-lysine–coated glass coverslips inverted over a glial feeder layer after 2-hour incubation. Cells were cultured at low density (2700 cells/cm^2^) so that axons and dendrites from individual neurons did not overlap. Paraffin dots attached to the coverslips kept neurons separated from the glial feeder cells. Neurons were subsequently processed for TEM between 1 and 2 days in vitro (DIV). All neurons were maintained in a humidified 37°C incubator with 5% CO_2_.

#### Primary fibroblast culture

We obtained skin fibroblasts from a healthy human individual as described previously (gift of M. Hirano, Columbia University) (*76*, *77*). Skin biopsy to obtain the fibroblast cells was only performed following informed consent, and all studies were performed in accordance with a Columbia University Medical Center Institutional Review Board–approved protocol (#AAAJ8651). Fibroblasts were cultured in DMEM supplemented with 15% fetal bovine serum (Sigma-Aldrich), 1% vitamin solution, and 1% antibiotic-antimycotic solution (Thermo Fisher Scientific). All experiments were conducted on cells cultured for <15 passages.

### Live-cell imaging

#### STED microscopy

Live-cell STED microscopy was conducted on a Leica SP8 STED 3× microscope (Leica Microsystems, Wetzlar, Germany) equipped with a SuperK EXTREME EXW-12 pulsed white light laser (NKT Photonics Inc., Portland, OR) as an excitation source, a Katana-08HP pulsed depletion laser as a depletion light source (775 nm; NKT Photonics), and a Leica high-contrast plan apochromat 93× 1.30 numerical aperture (NA) glycerol CS2 objective with motorized correction collar (Leica Microsystems); imaging was controlled by Leica Application Suite X software (LAS X, Leica Microsystems). Cells were imaged in a temperature-controlled Tokai Hit microscope stage top incubator (Amuza Inc., San Diego, CA) set to 37°C with 5% CO_2_. mNeon-KDEL was imaged with 505-nm excitation and 775-nm depletion wavelengths. The detection gate was set at 0.3 to 6 ns with a hybrid detector. Forty-nanometer pixel size was chosen on the basis of the Nyquist sampling criterion. All live-cell image stacks were acquired with 2× line averaging and 0.1-μm z-steps with 30 ms per frame. Image deconvolution was performed to remove noise via the classic maximum likelihood estimation deconvolution algorithm using Huygens Professional software (version 16.10, Scientific Volume Imaging, Hilversum, the Netherlands) as described previously (*78*). Deconvolution of the original raw image data was limited to 20 iterations of processing.

#### HiLo microscopy

HiLo microscopy was conducted on a Nikon TiE fully motorized inverted microscope equipped with a 100× 1.49 NA TIRF lens. Illumination used a 488-nm laser at an incident angle that allowed the lower ~0.5 μm of the cell to be imaged. Images were collected using a 3-mm stress-free dichroic with a 488-nm notch reflection and a 505/20-nm emission filter. Images were collected with a Photometrics 95B backthinned complementary metal-oxide semiconductor (CMOS) camera at 10 frames/s using NIS-Elements (version 5.2). All images were collected from samples mounted in a Tokai Hit environmental chamber set to 37°C with 5% CO_2_. Subsequent image analysis included a modified Richardson-Lucy deconvolution step adapted to work on 2D images (with a predicted point spread function). No other processing was performed to images. Kymographs of the images were generated using NIS-Elements. Colocalization of mNeon-KDEL and Halo-Sec61β signals was quantified via NIS-Elements by first applying the Richardson-Lucy deconvolution algorithm, followed by subsequent segmentation to accurately define positive signal for both channels. We then determined the percentage of the signal from the 640-nm channel (Halo-Sec61β) that colocalized to the 488-nm channel (mNeon-KDEL).

#### High-speed wide-field 3D microscopy

High-speed wide-field 3D images were acquired on a Nikon TiE microscope equipped with a 100× 1.49 NA TIRF objective and a Lumencor Spectra X (470-nm illumination) Photometrics 95B scientific CMOS (sCMOS) camera (Lumencor Inc., Beaverton, OR). Samples were mounted in a Tokai Hit environmental chamber (37°C with 5% CO_2_) and imaged as 3D stacks (20 frames/s, 0.8 s per stack,12-ms exposure, 15 *z* positions, 0.3-μm separation). An extended depth of focus projection was generated for each time point. No other processing was performed to images. During imaging of neurons, low magnification of the cells was used to distinguish neuronal axons from dendrites based on their established distinctive morphologies (e.g., identification of the axon initial segment that gives rise to the axon). Once identified, images of dendrites were subsequently acquired. Kymographs of the images were generated using NIS-Elements.

#### Cell viability imaging

INS-1E cells were seeded onto fibronectin-coated QUANTIFOIL gold London-finder EM grids with the grids placed into 35-mm-diameter circular glass-bottom dishes (MatTek). Cells were cultured for 48 hours under standard conditions in complete, serum-containing RMPI 1640 medium. To determine whether cells remained viable following this period, the grid-associated cells were labeled using the fluorescence-based LIVE/DEAD cell viability assay (Thermo Fisher Scientific) according to the manufacturer’s instructions. Samples were then mounted in a Tokai Hit environmental chamber (37°C with 5% CO_2_) and imaged by confocal microscopy using a Nikon Eclipse Ti imaging system equipped with a 1.40 NA 60× objective lens and swept field confocal scan head (Prairie Instruments, Madison, WI). For overnight imaging of cells grown on cryo-EM grids, dishes containing the grids were imaged in a humidified 37°C environmental chamber in the presence of a 20% O_2_, 5% CO_2_, and nitrogen-balanced gas mixture. Following confocal fluorescent imaging, cells were imaged using DIC microscopy overnight at 30-min intervals.

#### Cell preparation and transfection

INS-1E cells were plated onto poly-d-lysine–coated 35-mm circular glass-bottom dishes (MatTek) at a density of 4 × 10^5^ cells per dish and cultured for 48 hours in complete, serum-containing RPMI 1640 medium (37°C with 5% CO_2_). Cells were transfected with respective DNA constructs using Lipofectamine 3000 (Invitrogen) according to the manufacturer’s instructions. Imaging occurred 14 to 18 hours after transfection in either complete serum-supplemented RPMI 1640 medium (HiLo, wide-field, and DIC) or serum-supplemented FluoroBrite DMEM imaging medium (Thermo Fisher Scientific) (STED) to maintain optimal cell health throughout imaging. Primary rat cortical neurons were plated onto MatTek 35-mm circular glass-bottom dishes coated with poly-d-lysine and laminin following isolation (see above). Neurons were transfected at DIV7 with Lipofectamine 3000 and imaged 14 hours later (DIV8) in complete culture medium. To label Halo-Sec61β–expressing cells, cells were incubated with 500 nM JF_646_-HaloTag ligand (in serum-supplemented complete medium) for 1 hour (37°C with 5% CO_2_), washed extensively, and placed into complete serum-supplemented RPMI 1640 culture medium for imaging.

### EM grid preparation

#### Cryo-EM and cryo-ET

INS-1E cells were plated onto either fibronectin-coated 200 mesh gold R2/1 QUANTIFOIL grids or 200 mesh gold R2/2 London finder QUANTIFOIL grids (Quantifoil Micro Tools GmbH, Jena, Germany) at a density of 2 × 10^5^ cells/ml. Cells were cultured on the grids for 48 hours under conventional culture conditions in complete, serum-containing RPMI 1640 medium as described earlier (*45*). Grids were then removed directly from culture medium and immediately plunge-frozen in liquid ethane using a Vitrobot Mark IV (Thermo Fisher Scientific, FEI, Hillsboro, OR). Similarly, for all other cell types imaged by cryo-EM and cryo-ET including MEFs and BE(2)-M17 cells, cells were plunge-frozen immediately after being removed from their respective complete culture medium; a subset of MEF samples were exposed to tunicamycin for induction of ER-mitochondrial MAM contacts before plunge-freezing. For cell transfections of grid-grown cells, INS-1E cells were plated onto fibronectin-coated gold QUANTIFOIL grids and cultured for 24 to 48 hours in complete, serum-containing RPMI 1640 medium under standard culture conditions (37°C with 5% CO_2_). The cells were then transfected with the respective DNA constructs using Lipofectamine 2000 (Life Technologies) according to the manufacturer’s instructions. The cells were plunge frozen following overnight incubation in complete, serum-containing RPMI 1640 medium.

#### CSTET

WI-38 cells and HDMECs were grown on EM grids as described previously (*44*). Briefly, cells were plated on gold QUANTIFOIL grids and grown to 30 to 70% confluence, typically over 2 to 3 days. The cells were subsequently vitrified in liquid ethane using a Leica EM GP plunger and stored in liquid nitrogen until use.

### Cryo–electron microscopy

Cryo-EM images of INS-1E cells were recorded at ×10,000 and ×12,000 magnifications at 2× binning (21.7 and 18.1 Å, respectively) on the JEOL3200 FSC electron microscope (JEOL Ltd., Tokyo, Japan) at 300 kV using an in-column omega energy filter with zero-loss peak set to a slit width of 20 eV. Large cell areas were mapped as montages using SerialEM (Boulder Laboratory for 3-D Electron Microscopy of Cells, Boulder, CO). Images were recorded on a Gatan US4000 charge-coupled device (CCD) camera at 2× binning for improved contrast (Gatan Inc., Pleasanton, CA). Tomographic tilt series for INS-1E, MEF, and primary human fibroblast cells were recorded on a FEI Polara F30 electron microscope (Thermo Fisher Scientific-FEI) at 300 kV with a tilt range of ±60° in 1.5° increments using the Gatan K2 Summit direct detector (Gatan Inc.) in super-resolution mode at 2× binning to 2.6 Å per pixel; tilt series were acquired via SerialEM. Tomographic tilt series for MEFs were collected on a FEI G2 Polara 300-kV field emission gun (FEG) TEM equipped with an energy filter (slit width, 20 eV; Gatan Inc.) with a tilt range of ±60° in 1° increments and a 4k × 4k K2 Summit camera (Gatan Inc.) in counting mode at 2.6 Å per pixel. For BE(2)-M17 cells, tomographic tilt series were recorded at ×10,000 magnification on the JEOL3200 FSC electron microscope at 300 kV. Tilt series were collected with a tilt range of ±60° with 2° increments using the Direct Electron DE20 detector (Direct Electron LP, San Diego, CA) at 7.4 Å per pixel using SerialEM. The cumulative dose of each tilt series was 80 to 100 *e*^−^/Å^2^. Tilt series were subsequently aligned and reconstructed using the IMOD software package (*79*).

### Cryo-correlated light and electron microscopy

INS-lE cells were grown on 200 mesh gold R2/2 London finder QUANTIFOIL grids (Quantifoil Micro Tools GmbH) coated with fibronectin and plunge-frozen in liquid ethane using an FEI Vitrobot Mark IV (Thermo Fisher Scientific, FE). Immediately before plunge-freezing, 3 μl of a fluorescent microsphere/gold solution was applied to grids. Five hundred–nanometer (345/435 nm) blue polystyrene fluoropheres (Phosphorex Inc., Hopkinton, MA) were diluted in phosphate-buffered saline (PBS) and mixed with 20-nm colloidal gold (Sigma-Aldrich) pretreated with bovine serum albumin. This combination of blue fluorospheres and 20-nm colloidal gold was used as fiducial markers. Specifically, 20-nm colloidal gold was used during cryo-tomogram reconstruction, while the 500-nm blue fluorescent beads, with detectable fluorescent emission in both blue and green channels, were used along with the centroids of autofluorescent puncta to align all fluorescent images. The 500-nm blue fluorospheres also served as landmarks to map the location of target areas based on phase-contrast and low-magnification cryo-EM images and also served as reference points to correlate the fluorescent light microscope (FLM) and cryo-EM images. Frozen grids were subsequently loaded into Polara EM cartridges, transferred into a cryo-FLM stage [FEI Cryostage (*80*), modified to hold Polara EM cartridges (*81*)] mounted on a Nikon Ti inverted microscope, and imaged using a 60× extra long working distance (ELWD) air objective (Nikon CFI S Plan Fluor ELWD 60× NA 0.7, WD 2.62 to 1.8 mm) and a Neo 5.5 sCMOS camera (Andor Technology, South Windsor, CT), with a real-time deconvolution module using NIS-Elements software. Following cryo–light microscopy imaging, EM cartridges containing frozen grids were stored in liquid nitrogen and maintained at ≤−150°C throughout, including transfer and imaging. Grids were imaged using an FEI G2 Polara 300 kV FEG TEM equipped with an energy filter (slit width, 20 eV; Gatan Inc.) and a 4k × 4k K2 Summit camera (Gatan Inc.) using electron counting mode. Pixels on the detector represented 2.6 Å (41,000×) at the specimen level. Tilt series were recorded with a tilt range of ±60° in 1° increments and 10-μm underfocus. The cumulative dose of a tilt series was 80 to 100 *e*^−^/Å^2^. UCSF Tomo (*82*) was used for automatic acquisition of the tilt series. The tilt series were aligned and binned fourfold into 1k × 1k arrays using the IMOD software package (*79*). Red and either green or yellow (depending on the fluorophore) FLM images were collected using 2-s exposures. To overlay the cryo-tomograms onto the FLM and phase-contrast images, low-magnification EM images of the target area were acquired to locate surrounding clusters of 500-nm blue fluoropheres. Phase-contrast and FLM images were then rescaled using Abode Photoshop CS6 software (Adobe Systems, San Jose, CA) to match the EM images based on 500-nm blue fluorospheres and landmarks including 2-μm QUANTIFOIL holes. The cryo-tomograms were overlaid and correlated with the epifluorescent image using features such as crystalline ice and the 2-μm-diameter EM grid holes seen in both the cryo-tomogram and low-magnification EM image to form the final composite image.

### Cryo-FIB milling

#### Specimen thinning

Cryo–light microscopy of cells expressing ER-localized PIS-GFP guided cryo-FIB milling to regions of interest in the *x*-*y* plane. Plunge-frozen grids were then mounted in custom-modified Polara cartridges with channels milled through the bottom. This allowed samples to be milled at a low angle of incidence (∼10° to 12°) with respect to the carbon surface. These modified cartridges were transferred into an FEI Versa 3D equipped with a Quorum PP3010T Cryo-FIB/SEM preparation system (Quorum Technologies LLC, East Sussex, UK). The stage temperature was held at −183°C for all subsequent steps. Samples were sputter coated with 20 nm of platinum before milling to minimize curtaining and to protect the front edge of the sample during milling. Vitrified cells lying approximately perpendicular to the focused ion beam were located via SEM, and lamellae (∼12 μm wide and ∼2 μm thick) were rough milled with beam settings of 30 kV and 0.300 nA. A polishing mill at a reduced current of 30 pA was then performed to bring the final thickness to 150 to 400 nm. Samples were removed from the cryo-FIB/SEM while maintaining a temperature below −160°C and stored in liquid nitrogen until use (*83*).

#### Cryo-ET of cryo-FIB milled specimens

The cryo-EM grid, still in the modified Polara cartridge, was inserted into a FEI Polara F30 microscope operating at 300 kV equipped with an energy filter (slit width, 20 eV; Gatan Inc.) and a 4k × 4k K2 Summit direct detector using the direct electron counting mode (Gatan Inc.). Lamellae were located by making maps of the entire grid using SerialEM software (*84*). Pixels on the detector represented 6 nm (18,000×) at the specimen level. Tilt series were collected with a tilt range of ±60° in 1° increments and 4-μm underfocus using SerialEM software (*84*). The tilt series were aligned and binned by 4 into 1k × 1k using the IMOD software package (*79*), and 3D reconstructions were calculated using the simultaneous reconstruction technique implemented in the TOMO3D software package (*85*) or weighted back projection using IMOD. Cryo-tomograms were subsequently segmented using the Amira software package (Thermo Fisher Scientific, FEI). Segmentation was performed manually using density thresholds. Morphological measurements of segmented data were also performed in Amira. Movie image sequences were generated in JPEG format in Amira (Thermo Fisher Scientific, FEI) and converted into movies using QuickTime Player 7. Photoshop CS6 (Adobe) was then used to produce the final versions of the movies.

### Cryo–scanning transmission electron tomography

CSTET of WI-38 cells and HDMECs was performed as described previously (*44*). Briefly, bright-field STEM tomograms of both cell types were collected on a Tecnai F20 microscope (Thermo Fisher Scientific, FEI) at 200 kV, recorded in 2° increments between −60° and +60° tilts. Spatial sampling was set between 1 and 4 nm per pixel. Doses were limited to 1 to 3 *e*^−^/Å^2^.

### Computation

#### 2D image analyses

Images were analyzed in 3DMOD (*79*) (Boulder Laboratory for 3-D Electron Microscopy of Cells, Boulder, CO) and Fiji/ImageJ (NIH, Bethesda, MD). Montages were aligned and stitched using the SerialEM “blendmont” command. We used blinded raters for image analysis and used an in-house MATLAB script to establish identity of the respective organelles including secretory vesicles and RAVs based on a majority vote of the raters. Similarly, the intracellular distribution of organelles (including secretory vesicles, RAVs, and ribosomes) within the 2D images was analyzed using the IMOD software package based on established stereological approaches (*38*–*40*). Specifically, fields of defined areas were chosen in the cell body, protrusions, and protrusion tips; organelles within these respective areas were counted. Results were represented as the density of organelles per nm^2^ and calculated from at least five separate experiments.

Imaged 2D crystalline arrays within the secretory granule lumen were analyzed using the 2dx software package (*86*). Filtered images of crystalline arrays were created by masking the diffraction spots from the Fourier transforms.

#### Volume reconstruction, subtomogram extraction, alignment, and averaging

Alignment and weighted back-projection of tilt series were performed with SerialEM’s ETOMO program. Manual selection of subvolumes containing putative ribosomes was conducted on an 8× binned map using EMAN2’s Boxer program (*87*). Subtomograms were normalized, contrast-inverted, and placed into a Hierarchical Data Format (HDF) stack. To rule out reference bias, we conducted reference-free alignment and averaging of 3D subvolumes corresponding to the RAV membrane–bound particles using the ML_TOMO program within the Xmipp software package as previously described (*28*, *88*). Briefly, this program permits alignment and classification of 3D images with missing data regions in Fourier space by a 3D multireference refinement based on a maximum-likelihood target function, starting from random assignments of the orientations.

To estimate the resolution of the subtomogram average of RAV membrane–bound 80*S* ribosomes visualized in situ, we used the FSC to compare our subtomogram average with the subtomogram average of dog (*Canis lupus familiaris*) 80*S* ribosomes associated with pancreatic ER obtained by the Förster group (*26*) (EMD-3071). As the maps are entirely independent, we used a threshold of 0.143.

#### Analysis of subtomograms

The subtomograms containing putative RAV membrane–bound ribosomes were aligned separately according to the reference-free approach as described above. Image segmentation to delineate the RAV membrane was manually conducted using Amira software. The average distance between RAV-bound ribosomes was determined through in-house nearest-neighbor calculations using the coordinates of the segmented, bound ribosomes in Amira. Because of the missing wedge effect in cryo-ET, which leads to a loss of resolution in the *z* direction, the membranes shown in fig. S5 appear as barrels rather than spheres. The orientation of the protein exit tunnel with respect to the membrane was determined by aligning a high-resolution human 80*S* ribosome cryo-EM map where the location of the exit tunnel is known; the map was obtained from subtomogram averaging.

### Transmission electron microscopy

#### WI-38 cells

Human embryonic lung fibroblast–derived WI-38 cells were imaged by conventional TEM as described earlier (*44*). Briefly, cells were grown on glass coverslips, fixed with 2.5% glutaraldehyde and 2% paraformaldehyde (in 0.1 M sodium cacodylate buffer at 25°C), washed in cacodylate buffer (4°C), and stained with 1% osmium tetroxide and 2% uranyl acetate. Samples were dehydrated in cold ethanol and embedded in Epon. Upon removal of the glass coverslips with liquid nitrogen, ultrathin sections (~70 nm) were cut parallel to the coverslip surface and transferred to 200 mesh copper grids. Sections were imaged in a 120 kV FEI Tecnai Spirit T-12 equipped with a 2k Eagle CCD camera (Thermo Fisher Scientific, FEI).

#### SW-13 cells

TEM imaging of human adrenocortical SW-13 cells was described earlier (*89*). Briefly, cell monolayers were rinsed in PBS, fixed with 2.5% glutaraldehyde [in PBS (pH 7.4) for 1 h at 25°C), and postfixed in 1% osmium tetroxide with 1% potassium ferricyanide (for 1 hour at 4°C). Samples were dehydrated in ethanol and embedded in Epon. Ultrathin sections were cut, mounted on grids, and imaged on a JEOL 1011CX electron microscope (JEOL, Tokyo, Japan).

#### Primary hippocampal neurons

For TEM of primary hippocampal neurons, specimens were fixed in cold 2.5% glutaraldehyde (in 0.01 M PBS), postfixed in 1% osmium tetroxide with 1% potassium ferricyanide, washed in PBS, and dehydrated through a graded series of ethanol and propylene oxide steps. Samples were embedded in Poly/Bed 812 (Luft formulations, Polysciences Inc., Warrington, PA). Semithin sections (300 nm) were cut on a Leica Reichert Ultracut ultramicrotome and stained with 0.5% Toluidine blue (in 1% sodium borate). Ultrathin sections (65 nm) were subsequently stained with uranyl acetate and Reynold’s lead citrate and examined on a JEOL 1011 transmission electron microscope with a side mount AMT 2k digital camera (Advanced Microscopy Techniques, Danvers, MA).

### Metabolic analyses

INS-1E cells were seeded either into individual wells of a poly-l-lysine–coated Agilent Seahorse 96-well XF cell culture microplate (Agilent, Santa Clara, CA) or fibronectin-coated 200 mesh gold R2/2 London finder QUANTIFOIL grids at an initial cell density of 25,000 cells per well or grid. Cells were then grown for 48 hours under standard culture conditions in serum-supplemented RPMI 1640 medium (see above). The metabolic profiles, including determination of the OCR, of adherent cells either grown in the culture microplate or grown directly on the EM grids were subsequently determined using the Agilent XF96 Extracellular Flux Analyzer (Agilent). Cells under both conditions were placed into XF medium (RPMI 1640 medium containing 10 mM glucose, 4 mM l-glutamine, and 2 mM sodium pyruvate) to accurately measure OCR throughout a mitochondrial stress test as described previously (*90*). Briefly, the stress test consisted of initial baseline measurements, followed by sequential addition of oligomycin (5 μΜ), FCCP (10 μΜ), and rotenone (5 μΜ). To control for potential cell number variability between samples, all values were normalized to initial baseline OCR.

### ER stress assays

#### ER stress induction

ER stress induction was described previously (*91*, *92*). INS-1 cells, the parental cell line for INS-1E cells (gift of C. Wollheim, Université de Genève), were treated with either 1 μM thapsigargin (1 and 6 hours) or tunicamycin (2 μg/ml; 16 hours) to induce ER stress. At the indicated time points, cells were washed in PBS and lysed in ice-cold lysis buffer [1% Triton X-100, 20 mM Hepes, 100 mM KCl, 2 mM EDTA, 1 mM phenylmethylsulfonyl fluoride (PMSF), leupeptin (10 μg/ml), and aprotinin 10 μg/ml (pH 7.4)]. For experiments investigating protein phosphorylation, lysis buffer also contained phosphatase inhibitors (10 mM NaF, 2 mM Na_3_VO_4_, and 10 nM okadaic acid). The cells were lysed on ice for 30 min and centrifuged at 13,000 rpm (10 min at 4°C). The supernatant was then transferred to a new tube, and the protein concentration was determined with BCA reagent (Pierce Chemical Co., Rockford, IL).

#### Western blot analysis

Cell lysis and Western blot analysis were performed as described previously (*92*). Briefly, equal amounts of protein (20 μg) were run on SDS–polyacrylamide gel electrophoresis gels, transferred to nitrocellulose membranes, and immunoblotted with the following antibodies: p-eIF2α (1:500; #9721, Cell Signaling Technology, Danvers, MA), p-PERK (1:500; #3191, Cell Signaling), and growth arrest and DNA damage–inducible protein (GADD153)/CHOP (1:500; sc-575, Santa Cruz Biotechnology, Dallas, TX). Following incubation with secondary antibody conjugated to horseradish peroxidase, the respective bands were detected by enhanced chemiluminesence (RPN2106, Amersham Biosciences, Piscataway, NJ). Immunoblots were scanned via Scion Image software.

#### XBP1 splicing assay

The assay including primers and PCR conditions were described earlier (*91*). Briefly, total RNA was isolated using TRIzol Reagent (Invitrogen) from control untreated cells or cells treated with pharmacological inducers of ER stress, followed by purification with RNeasy Mini Kit (QIAGEN, Germantown, MD). Rat XBP-1 cDNA was amplified by RT-PCR (QIAGEN OneStep RT-PCR kit) using primers flanking the intron excised by IRE1 exonuclease.

### Statistical analyses

Statistical significance was determined using SPSS (version 18.0, IBM, Armonk, NY) and GraphPad Prism (version 5.0, GraphPad Software Inc., La Jolla, CA). Analyses included one-way analysis of variance (ANOVA) (α = 0.05) with a post hoc two-sided Dunnett *t* test for pairwise comparisons. No data were excluded from analyses.

## Supplementary Material

aay9572_Movie_S2.mp4

aay9572_Movie_S10.mov

aay9572_Movie_S1.mp4

aay9572_Movie_S4.mp4

aay9572_Movie_S11.mp4

aay9572_SM.pdf

aay9572_Movie_S3.mp4

aay9572_Movie_S9.mov

aay9572_Movie_S6.mp4

aay9572_Movie_S5.mp4

aay9572_Movie_S7.mp4

aay9572_Movie_S12.avi

aay9572_Movie_S13.avi

aay9572_Movie_S8.mov

## SUPPLEMENTARY MATERIALS

Supplementary material for this article is available at http://advances.sciencemag.org/cgi/content/full/6/14/eaay9572/DC1

## Appendix

View/request a protocol for this paper from *Bio-protocol*.
