# Supplementary material for: Ribosome-associated vesicles: A dynamic subcompartment of the endoplasmic reticulum in secretory cells
Source: Sci Adv. 2020 Apr 1;6(14):eaay9572. doi: 10.1126/sciadv.aay9572 (PMC7112762; doi:10.1126/sciadv.aay9572)
Supplement: aay9572_SM.pdf [file aay9572_SM.pdf]

Science Advances
AAAS

advances.sciencemag.org/cgi/content/full/6/14/eaay9572/DC1

Supplementary Materials for

Ribosome-associated vesicles: A dynamic subcompartment of the endoplasmic reticulum in secretory cells

Stephen D. Carter, Cheri M. Hampton, Robert Langlois, Roberto Melero, Zachary J. Farino, Michael J. Calderon, Wen Li, Callen T. Wallace, Ngoc Han Tran, Robert A. Grassucci, Stephanie E. Siegmund, Joshua Pemberton, Travis J. Morgenstern, Leanna Eisenman, Jenny I. Aguilar, Nili L. Greenberg, Elana S. Levy, Edward Yi, William G. Mitchell, William J. Rice, Christoph Wigge, Jyotsna Pilli, Emily W. George, Despoina Aslanoglou, Maïté Courel, Robin J. Freyberg, Jonathan A. Javitch, Zachary P. Wills, Estela Area-Gomez, Sruti Shiva, Francesca Bartolini, Allen Volchuk, Sandra A. Murray, Meir Aridor, Kenneth N. Fish, Peter Walter, Tamas Balla, Deborah Fass, Sharon G. Wolf, Simon C. Watkins, José María Carazo, Grant J. Jensen*, Joachim Frank*, Zachary Freyberg*

*Corresponding author. Email: freyberg@pitt.edu (Z.F.); jf2192@cumc.columbia.edu (J.F.); jensen@caltech.edu (G.J.J.)

Published 1 April 2020, *Sci. Adv.* **6**, eaay9572 (2020)
DOI: 10.1126/sciadv.aay9572

The PDF file includes:

Figs. S1 to S15

Other Supplementary Material for this manuscript includes the following:

(available at advances.sciencemag.org/cgi/content/full/6/14/eaay9572/DC1)

Movies S1 to S13

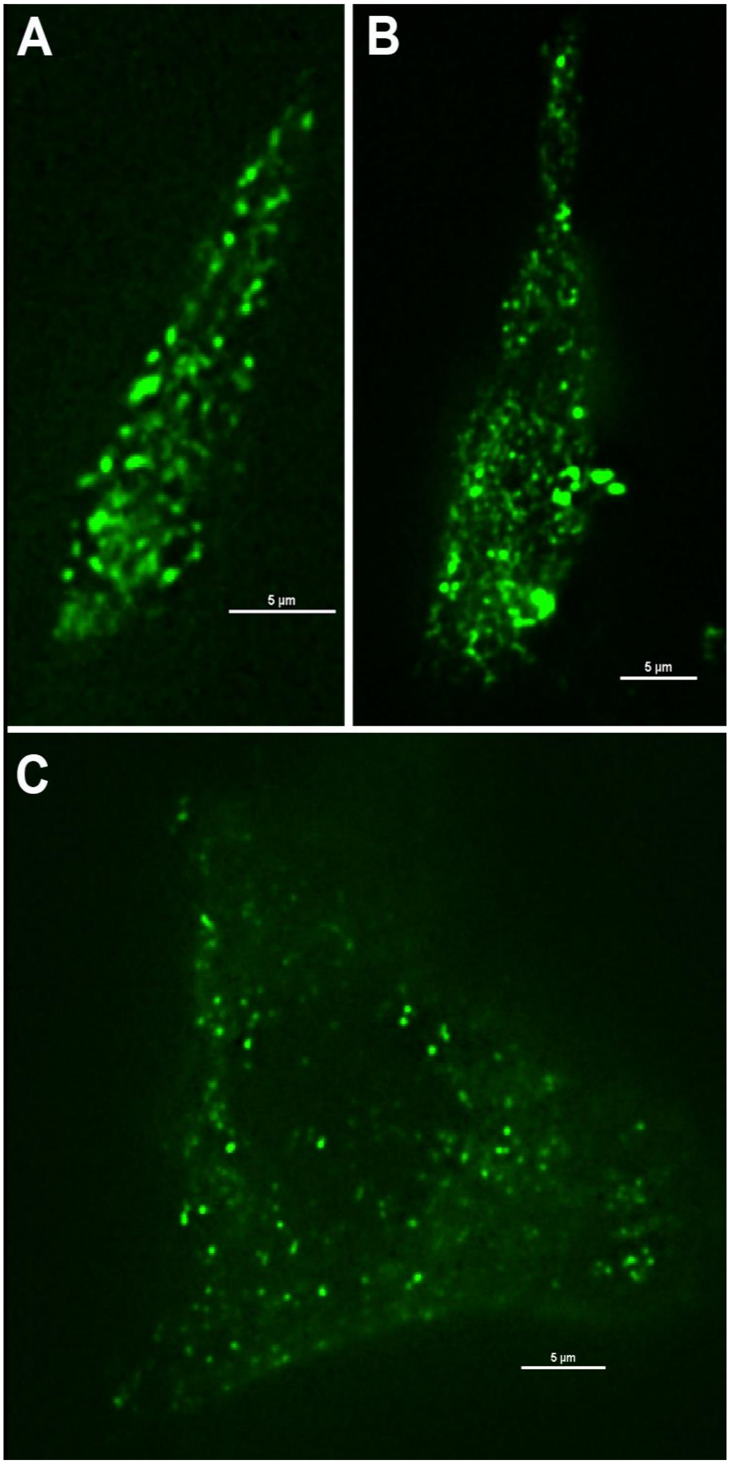

Fig. S1

Fig. S1. ER and dense core secretory granule markers localize to intracellular punctate structures. (A, B) Representative images of INS-1E cells expressing intraluminal ER markers calreticulin-EYFP (**Panel A**) and BiP-GFP (**Panel B**) and imaged via HiLo microscopy. Both ER markers localized to punctate structures distributed throughout the cell; scale bars = 5 μm. **(C)** Representative HiLo microscopy image of an INS-1E cell expressing dense core secretory granule marker CgA-GFP HiLo demonstrated a punctate expression pattern throughout the cytoplasm; scale bar = 5 μm.

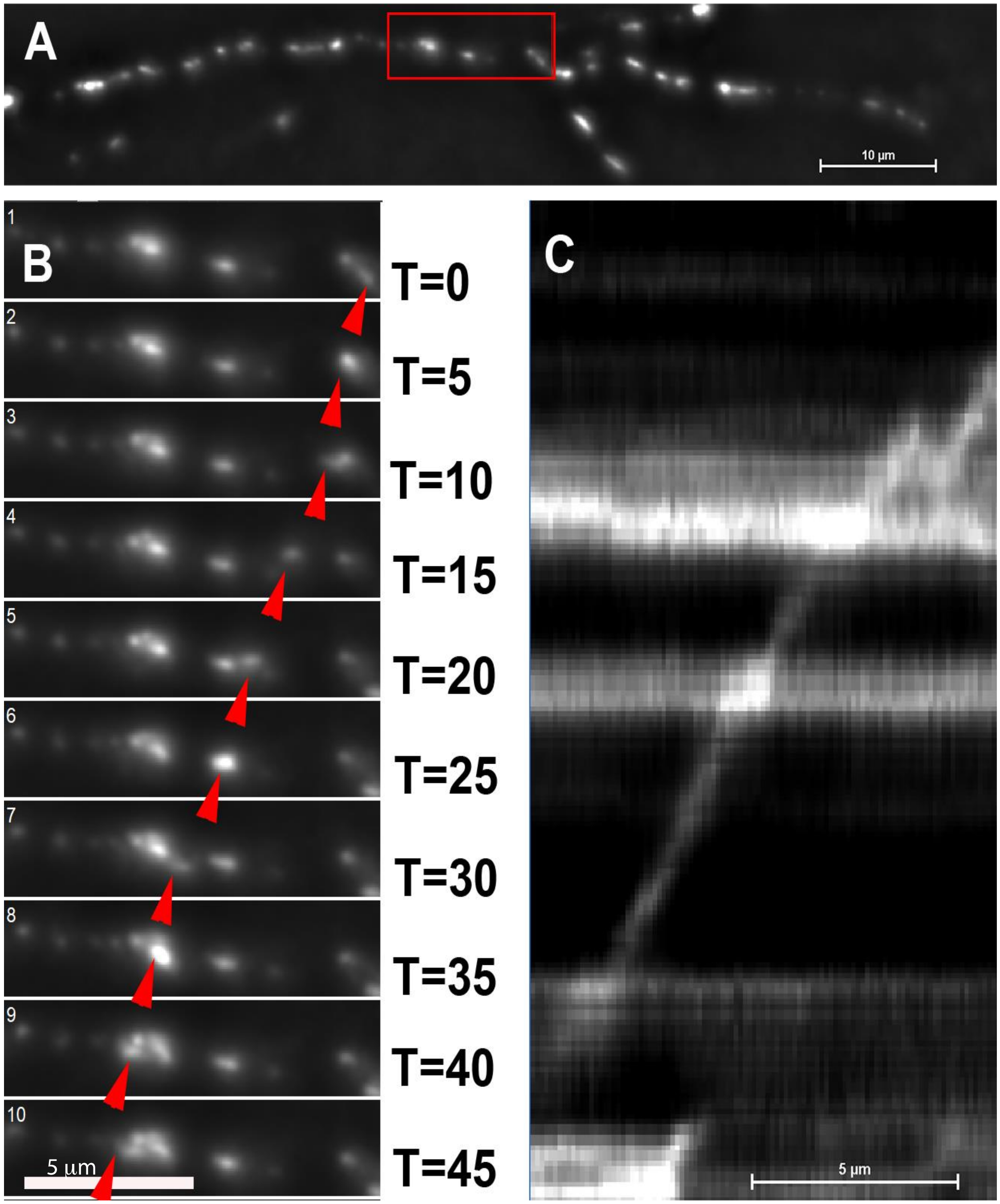
A
10 µm
B
1
2
3
4
5
6
7
8
9
10
5 µm
T=0
T=5
T=10
T=15
T=20
T=25
T=30
T=35
T=40
T=45
C
5 µm

Fig. S2

Fig. S2. Dynamic ER marker-labeled puncta in primary neuron dendrites. (**A**) High-speed widefield 3D imaging captured dynamic puncta labeled by ER luminal marker mNeon-KDEL in the dendrites of primary rat cortical neurons; scale bar = 10 μm. (**B**) Time lapse images show movement of a mNeon-KDEL-labeled punctum across space within the boxed region in **Panel A** at specific time points, as indicated by the red arrows (see **Movie S4** and **Movie S5**); scale bar = 5 μm. Images in **Panels A** and **B** are representative extended depth of focus projections. (**C**) Accompanying kymograph of motion across time for the punctum highlighted in **Panel B**; scale bar = 5 μm.

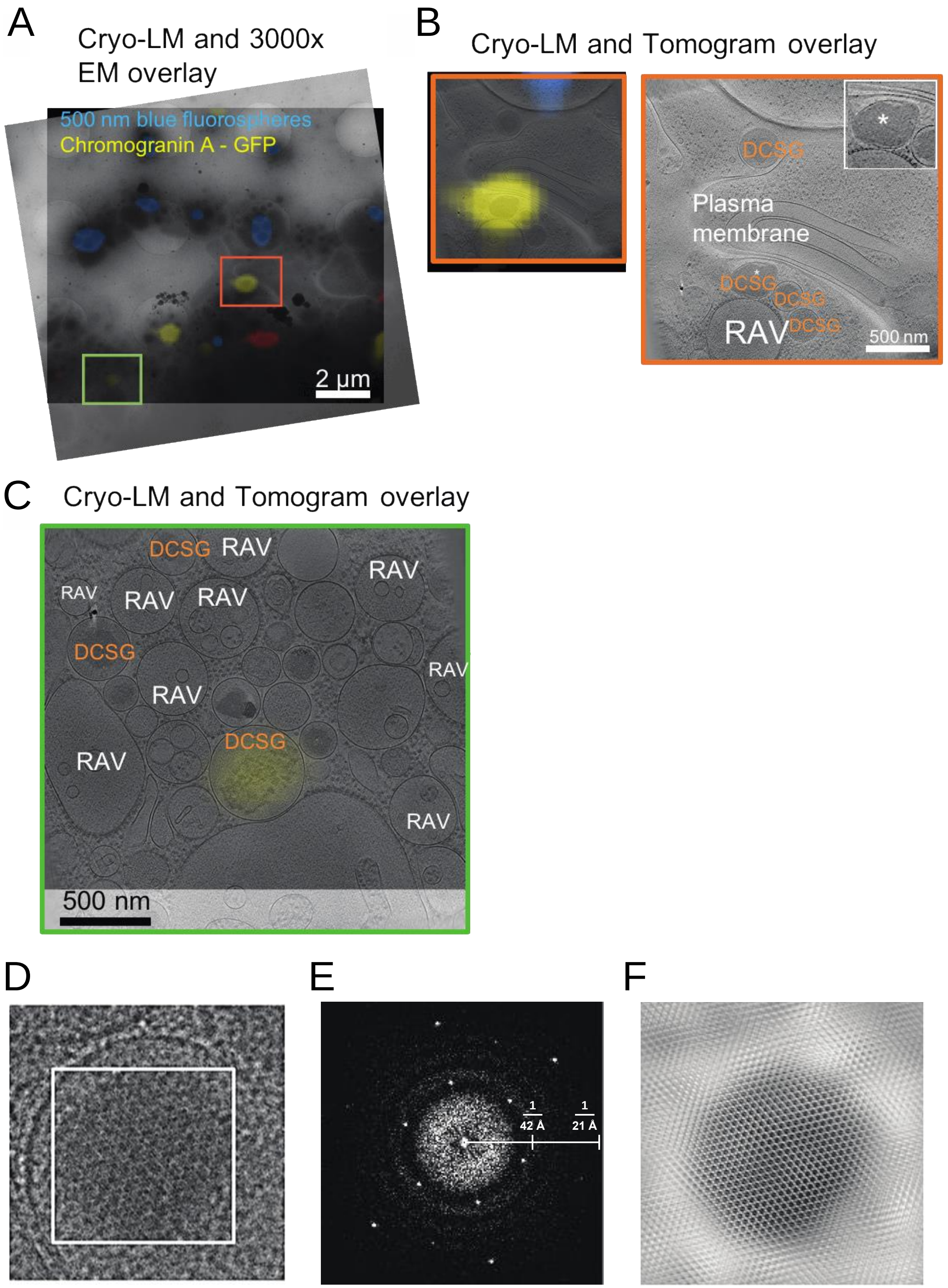
A
Cryo-LM and 3000x EM overlay
500 nm blue fluorospheres
Chromogranin A - GFP
2 µm
B
Cryo-LM and Tomogram overlay
DCSG
Plasma membrane
DCSG
DCSG
RAV
DCSG
500 nm
C
Cryo-LM and Tomogram overlay
DCSG
RAV
RAV
RAV
RAV
RAV
DCSG
RAV
RAV
DCSG
RAV
RAV
500 nm
D
E
1/42 Å
1/21 Å
F

Fig. S3

Fig. S3. Chromogranin A-GFP localizes to dense core secretory granules but not RAVs. (**A**) Cryo-CLEM in INS-1E cells expressing secretory granule marker chromogranin A-GFP (CgA-GFP). Epifluorescent images were correlated with a 3,000x magnification cryo-EM image to form the final composite image using clusters of 500 nm blue fluorospheres as fiducial markers; scale bar = 2 μm. (**B**) Enlarged view of the orange boxed area with tomographic slice and fluorescent image overlaid. CgA-GFP fluorescence localized to one dense core secretory granule (DCSG) lumen, but not in the RAV next to it. Alongside is an enlarged view of the tomographic slice, labeled with relevant subcellular structures and organelles; scale bar = 500 nm. Inset highlights a magnified view of a CgA-GFP-labeled DCSG with an apparent insulin crystal (labeled with an asterisk). (**C**) Enlarged view of the area boxed in green also demonstrating intracellular CgA-GFP localization. Scale bar = 500 nm. As in **Panel B**, CgA-GFP fluorescence localizes to one DCSG lumen, but not in RAVs or other vesicles. (**D**) We characterized the intraluminal crystalline arrays (arrow) identified within the secretory granule dense core, focusing on the boxed in area of the 2D cryo-EM image (white box, 200 pixels × 200 pixels). (**E**) From the crystalline array within the boxed-in area, we obtained the power spectrum which reveals 2D hexagonal symmetry; the resolution in inverse Å is indicated by the scale bars. (**F**) Altered image of the array was constructed by masking the diffraction spots in **Panel E**, revealing a unit cell size of a=56 Å, b=56 Å, γ=119°. Pixels on the detector represented 2.6 Å at the specimen level; images are non-montaged. Images in **Panels A-F** are representative of n>3 independent experiments.

A

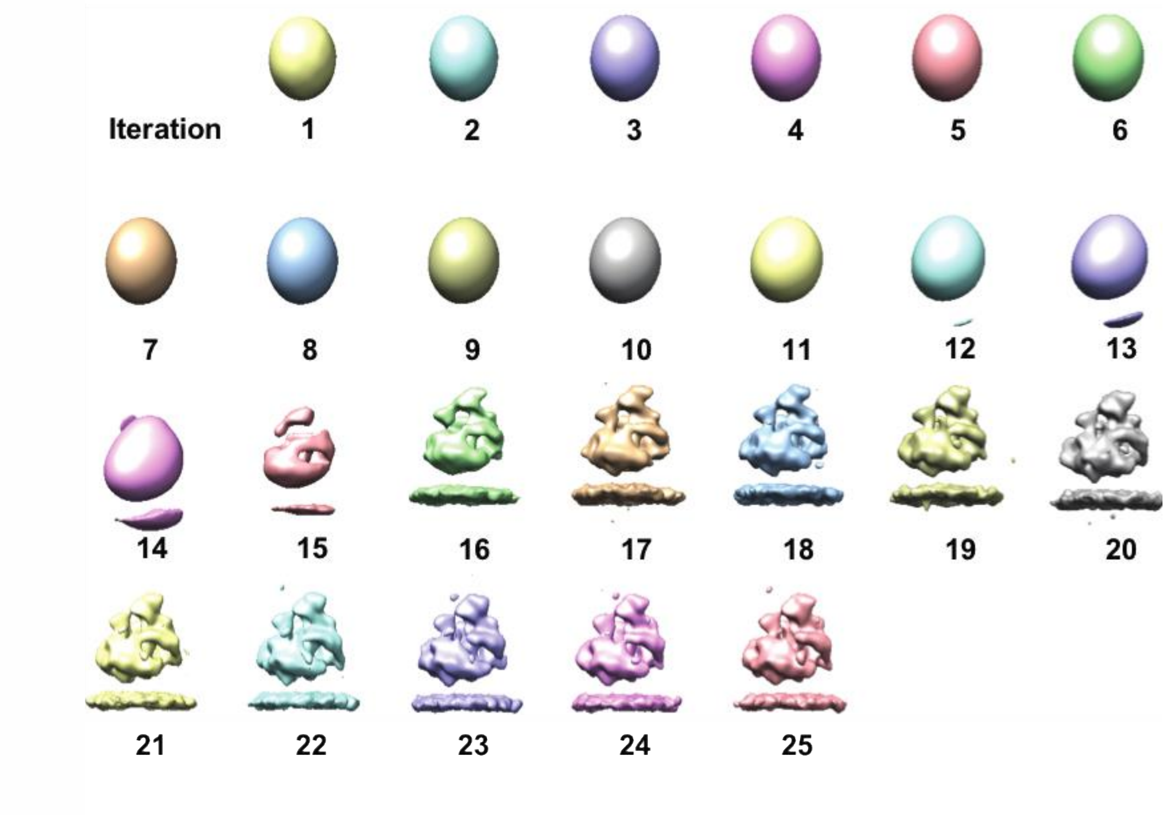

B

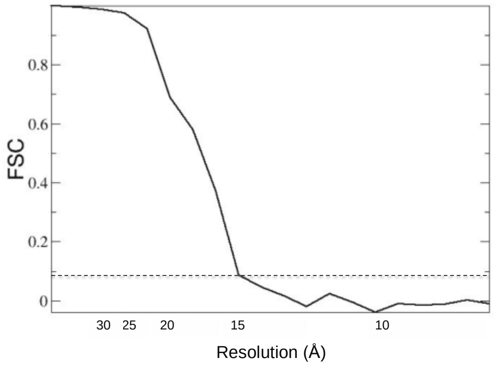

Fig. S4

Fig. S4. Validation of the ribosomal identity of RAV membrane-bound electron-dense particles using a reference-free approach. (**A**) To confirm ribosome identity while avoiding

reference bias, we used reference-free alignment and averaging of subtomograms containing electron-dense RAV-bound particles located *in situ.* The convergence of this iterative procedure is demonstrated here with a subset of 319 subtomograms. Over the course of successive iterations, beginning from random assignments of the orientations, we observe the progression of the corresponding subtomogram averages from a sphere to a shape that closely resembles reconstructions of the 80S mammalian ribosome. (**B**) Fourier Shell Correlation (FSC) plot comparing our subtomogram average with the with a human 80S ribosome map generated from an atomic model (*31*) (PDB 4UG0) yielded a 15 Å resolution for our subtomogram average using the FSC=0.143 criterion.

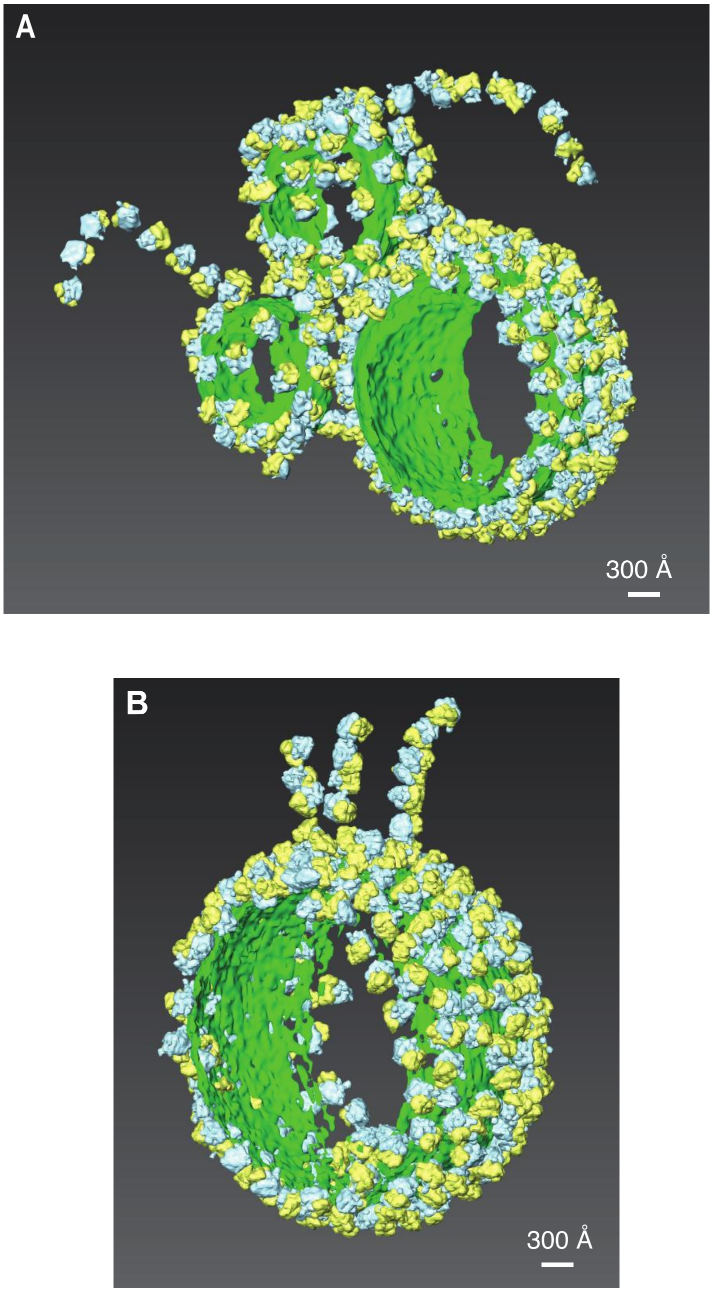

Fig. S5

Fig. S5. Polysome arrangement of ribosomes on RAVs. Polysomes associate with RAV membranes. Panels **A** and **B** are from two separate tilt series. Ribosome averages were

constructed from 254 and 235 ribosomes, respectively. The illustration was created by mapping a subtomogram average into original locations of the subtomograms containing putative ribosomes. Chains of roughly equally-spaced (average distance 320±11 Å) individual ribosomes approach the vesicles and appear to be continuous with linear arrangements of ribosomes bound on the membrane. Scale bars = 300 Å; yellow, 40S subunits; blue, 60S subunits; green, RAV membrane. Images are representative of n>3 independent experiments.

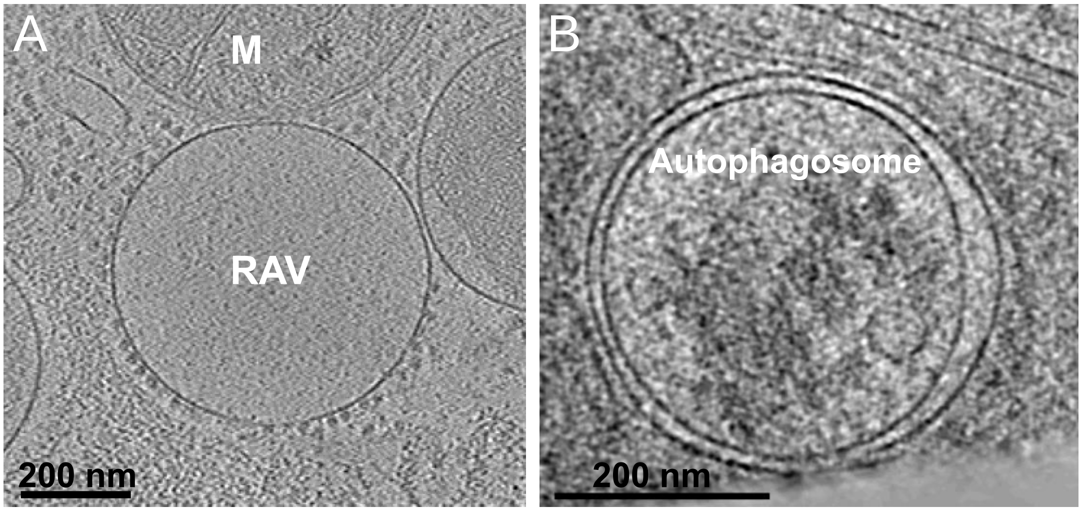

Fig. S6

Fig. S6. Comparison between RAV and autophagosome morphology. The morphology of a RAV features a single outer membrane studded with ribosomes (**Panel A**), which significantly differs from that of an autophagosome featuring a double membrane and no ribosomal association (**Panel B**). The respective structures are taken from single x-y slices of tomograms

acquired from a tilt-series of INS-1E cells; all scale bars = 200 nm. Pixels on the detector represented 2.6 Å at the specimen level; images are montaged in the overview with non-montaged images in the remaining panels.

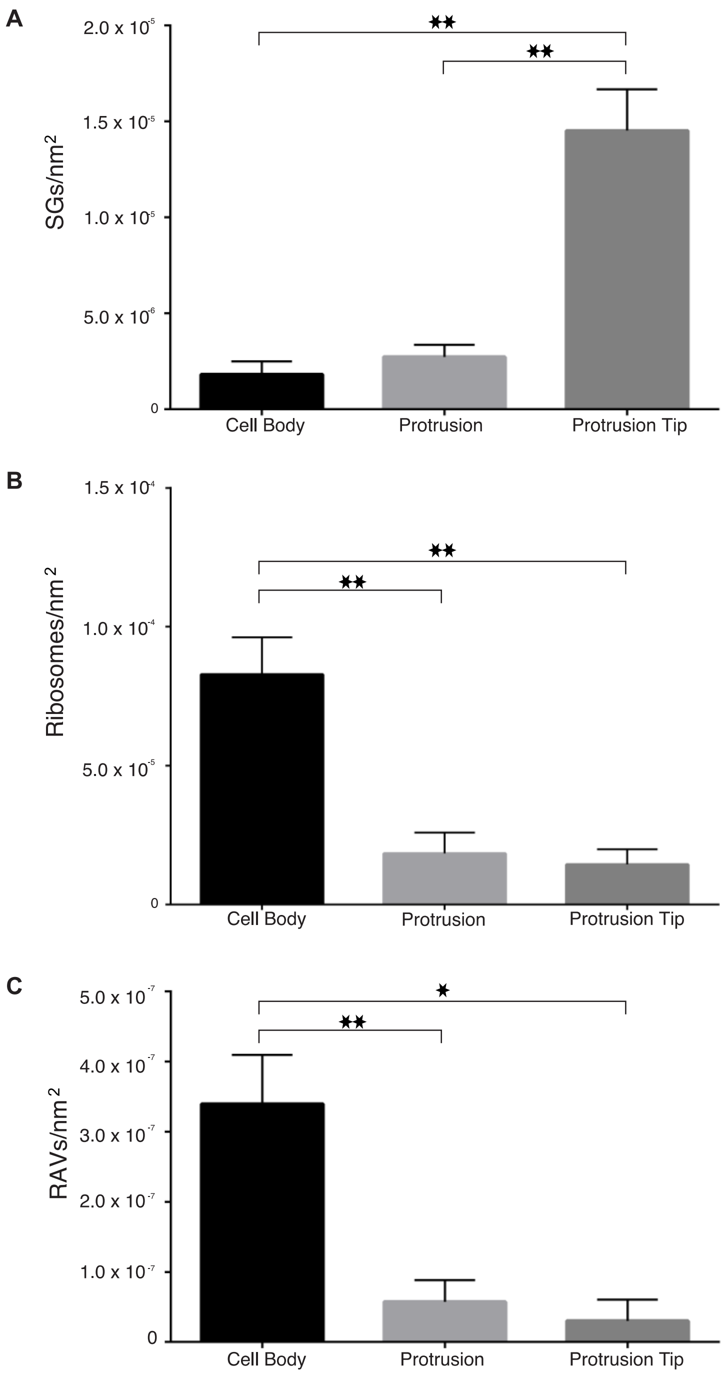
A
B
C
SGs/nm²
Ribosomes/nm²
RAVs/nm²
2.0 x 10-5
1.5 x 10-5
1.0 x 10-5
5.0 x 10-6
1.5 x 10-4
1.0 x 10-4
5.0 x 10-5
5.0 x 10-7
4.0 x 10-7
3.0 x 10-7
2.0 x 10-7
1.0 x 10-7
0
Cell Body
Protrusion
Protrusion Tip

Fig. S7

Fig. S7. Analysis of intracellular organelle distributions. (**A**) The intracellular distribution of secretory granules (SGs) was determined by cell region (cell body, protrusion, and protrusion tips) in INS-1E cells imaged by cryo-EM. The density of secretory granules (organelles per nm^2) was significantly greater in the cell protrusions and their tips compared to the cell body (P<0.0001; n=5). (**B**) Free, unbound ribosomes were significantly more densely concentrated in the cell body compared to cell protrusions and their tips (P<0.0001; n=6). (**C**) The intracellular distribution of RAVs mirrored free ribosomes, with the highest density in the cell body compared to the protrusions or tips (P=0.0005; n=7); error bars are represented as s.e.m.

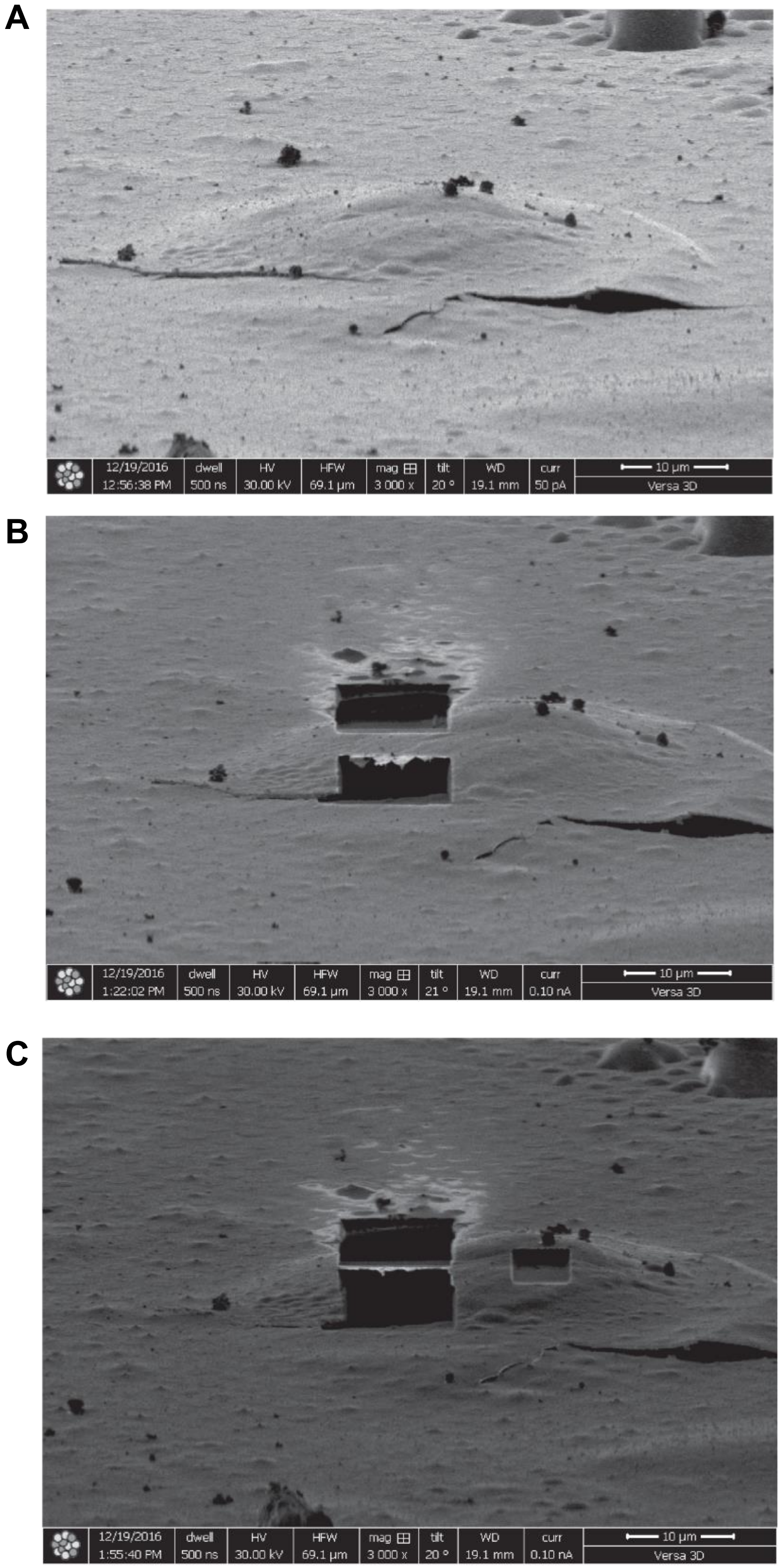
A
12/19/2016
12:56:38 PM
dwell
500 ns
HV
30.00 kV
HFW
69.1 µm
mag
3 000 x
tilt
20 °
WD
19.1 mm
curr
50 pA
10 µm
Versa 3D
B
12/19/2016
1:22:02 PM
dwell
500 ns
HV
30.00 kV
HFW
69.1 µm
mag
3 000 x
tilt
21 °
WD
19.1 mm
curr
0.10 nA
10 µm
Versa 3D
C
12/19/2016
1:55:40 PM
dwell
500 ns
HV
30.00 kV
HFW
69.1 µm
mag
3 000 x
tilt
20 °
WD
19.1 mm
curr
0.10 nA
10 µm
Versa 3D

Fig. S8

Fig. S8. Stages of cryo-focused-ion-beam milling of INS-1E cells. (**A**) INS-1E cells grown on Quantifoil cryo-EM grids, rapidly vitrified by plunge-freezing, coated with a protective platinum outer layer and subsequently visualized by cryo-SEM. (**B**) Areas for FIB-milling were selected on both sides of the cell using a dual-beam FIB/SEM equipped with a cryo-cooled stage and shuttle to hold the frozen grid. (**C**) Successive cryo-FIB-milling on both sides of the cell progressively generated a thin lamella with a thickness of ~100-300 nm (indicated by now-darkened boxed regions). Scale bars = 10 μm.

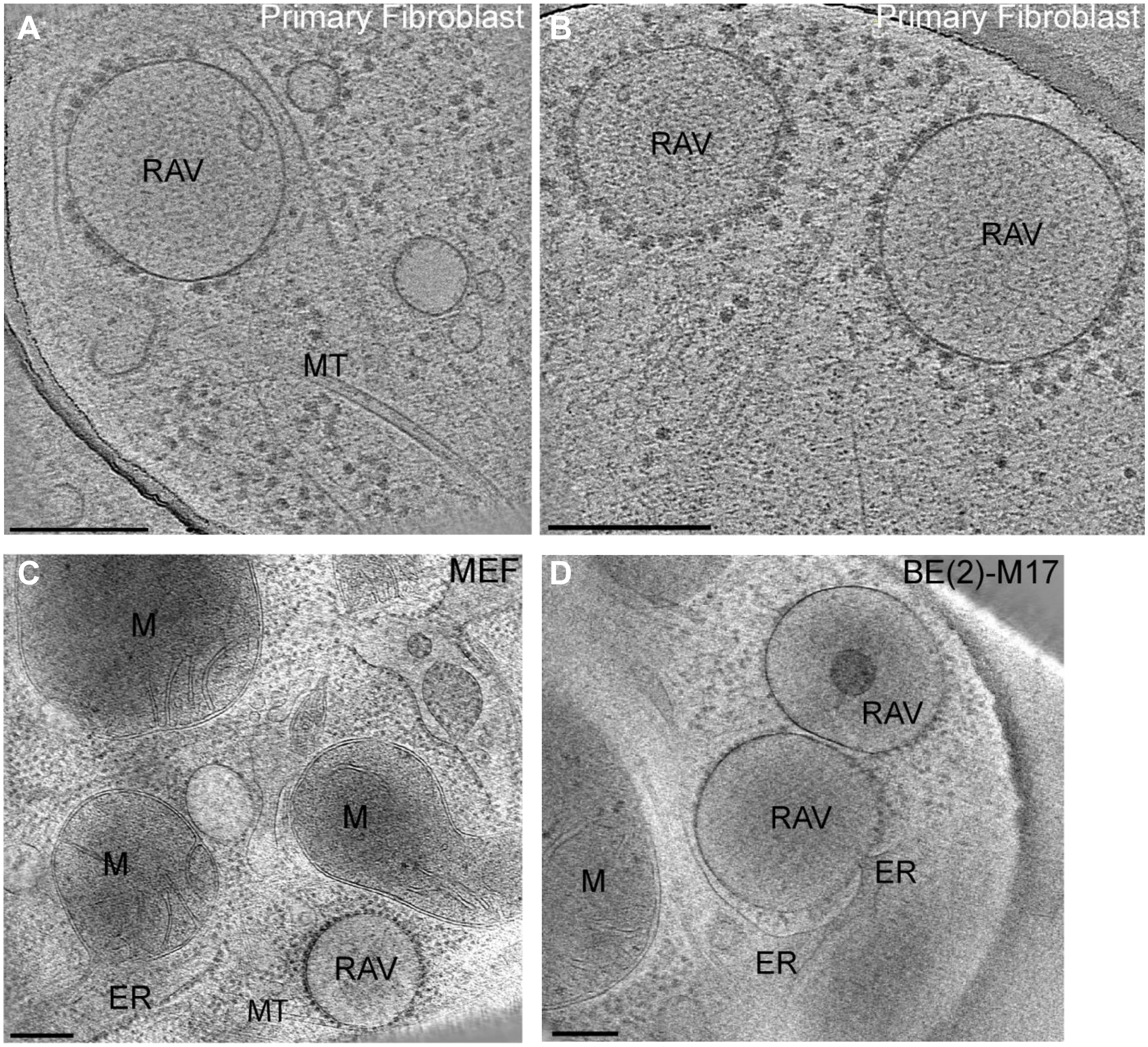

Fig. S9

Fig. S9. RAVs are present in multiple cell types by cryo-ET. RAVs were evident in three-dimensional cryo-electron tomograms of several cell types in addition to INS-1E cells. (**A**, **B**) We found several discrete examples of RAVs within primary human fibroblasts including alongside microtubules (MT). (**C**) RAVs were also observed in mouse embryonic fibroblasts (MEFs); and in (**D**) human dopamine neuron-derived BE(2)-M17 cells. Note the presence of the

ER network (labeled as ER) and mitochondria (labeled as M) alongside RAVs in **Panels C** and **D**. All imaging was conducted in untransfected cells cultured according to their respective standard culture conditions. Images represent tomogram x-y slices acquired from the respective tilt-series. All scale bars = 250 nm.

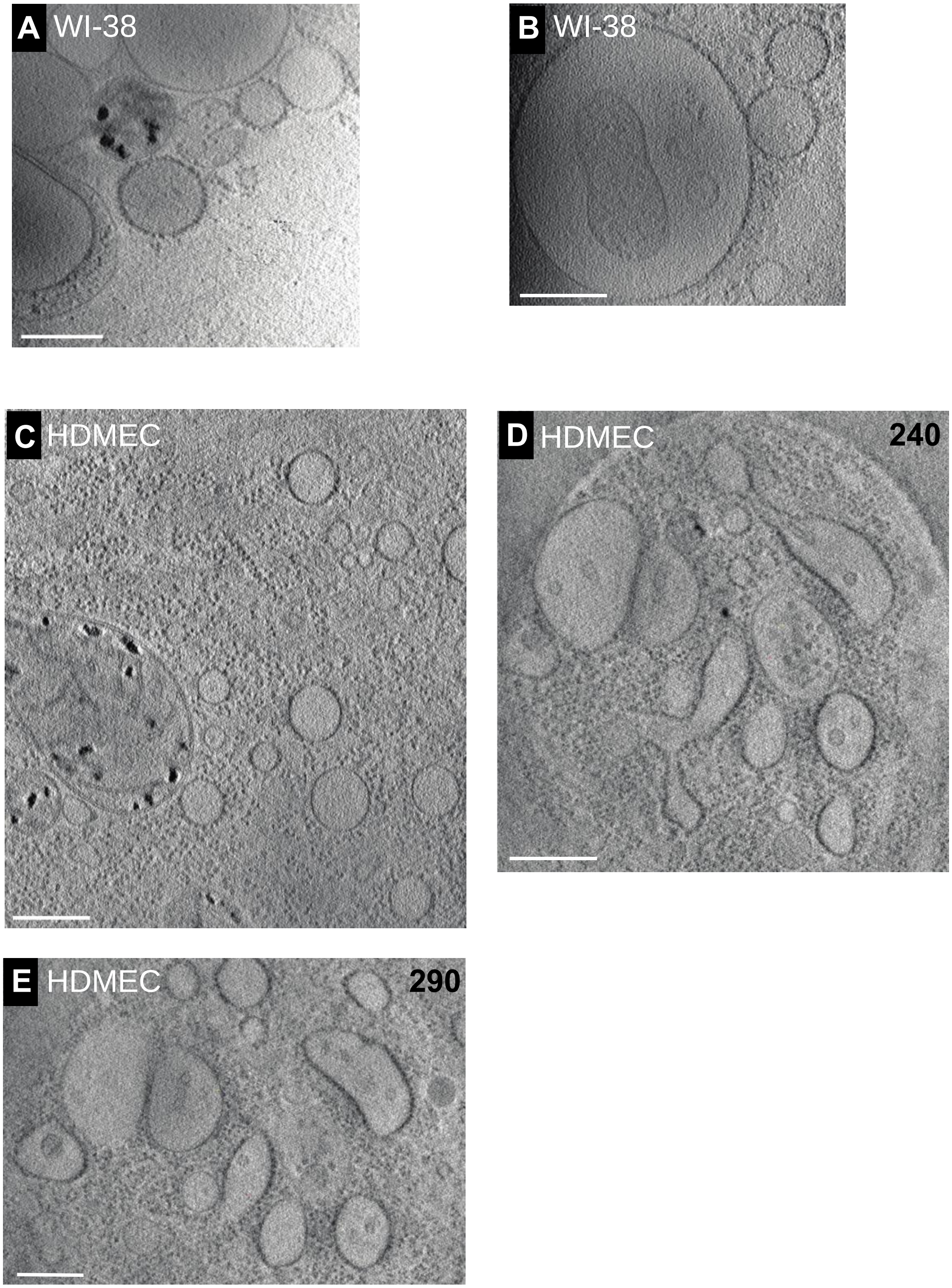

Fig. S10

Fig. S10. Thin virtual slices from reconstructions of CSTET brightfield cryo-tomograms. (**A**, **B**) CSTET imaging of untreated, untransfected human lung fibroblast WI-38 cells, and **(C)** untreated, untransfected human dermal microvascular endothelial cells (HDMEC) demonstrated RAVs in both cell types. (**D**, **E**) Views of RAVs within HDMEC cells at two different cell depths. Heights of the respective optical slices relative to the bottom of the cell are indicated. The overall cell thickness in the region where RAVs were visualized was 880 nm. All scale bars = 400 nm.

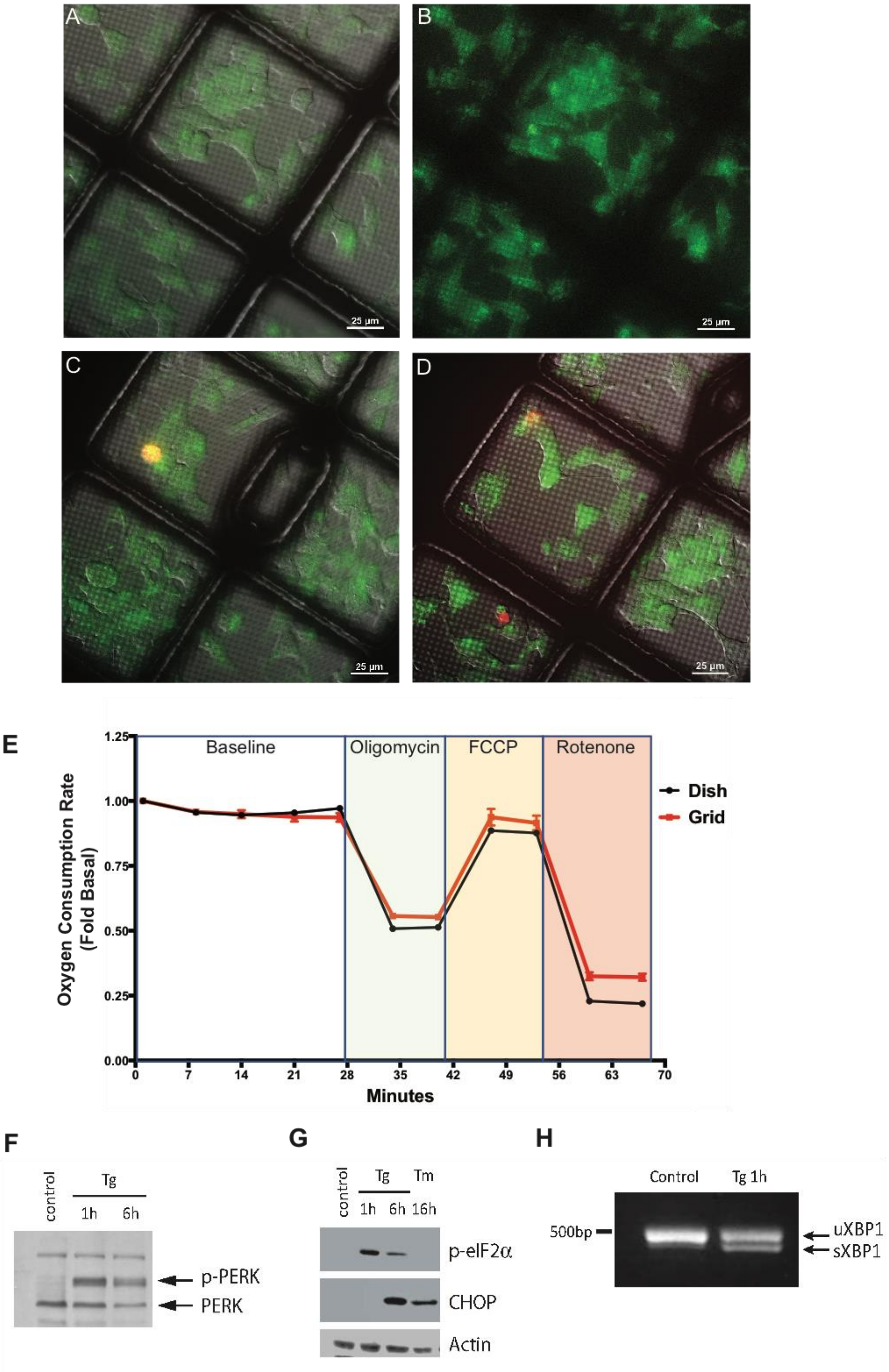
A
25 µm
B
25 µm
C
25 µm
D
25 µm
E
Baseline
Oligomycin
FCCP
Rotenone
Dish
Grid
Oxygen Consumption Rate (Fold Basal)
1.25
1.00
0.75
0.50
0.25
0.00
0
7
14
21
28
35
42
49
56
63
70
Minutes
F
control
Tg
1h
6h
p-PERK
PERK
G
control
Tg
Tm
1h
6h
16h
p-eIF2α
CHOP
Actin
H
Control
Tg 1h
500bp
uXBP1
sXBP1

Fig. S11

Fig. S11. Analysis of cell viability, energy metabolism and ER stress in INS-1E cells grown on EM grids. (**A-D**) INS-1E cells were grown on fibronectin-coated Quantifoil gold London-finder EM grids, labeled using the fluorescence-based LIVE/DEAD assay, and live-imaged by widefield fluorescence and DIC microscopy (see Methods) to determine cell viability and potential cytotoxicity related to growth on EM grids. Cells labeled with green fluorescent calcein-AM viability dye indicated living cells while red fluorescent ethidium homodimer-1 labeling indicated cytotoxicity and/or cell death. Panels **A-D** indicate representative fields from four separate EM grids; scale bars = 25 μm. In all imaged fields, >99% of cells were labeled with the vital green dye with only a small minority of detached, red fluorescent cells, suggesting that cells grown on EM grids were viable with little signs of cytotoxicity. (**E**) Energy metabolism in INS-1E cells grown directly on EM grids was analyzed. Mitochondrial oxygen consumption rates (OCR) were measured via Seahorse extracellular flux analysis in INS-1E cells grown either on EM grids under the same growth conditions used for our cryo-EM and -ET experiments (red square, n=8) or in 96-well tissue culture dishes (black circle, n=77). No significant differences in basal respiration measurements under conventional growth conditions were observed between cells grown on grids versus in culture dishes (P>0.05). Grid-grown cells responded comparably to dish-grown cells in response to subsequent sequential addition of inhibitors targeting different components of the mitochondrial respiration: oligomycin (5 μM), FCCP (10 μM), and rotenone (5 μM). To control for potential cell number variability between samples, all values were normalized to initial baseline OCR. Results shown represent the means ± s.e.m. for n>3 independent experiments. (**F-H**) Potential ER stress was examined in cells grown on EM grids. INS-1 cells, the parental cell line for INS-1E cells, were cultured under standard conditions as described in the Methods (control cells) or were treated for the indicated times with

pharmacological inducers of ER stress (1 μM thapsigargin, Tg; or 2 μg/ml tunicamycin, Tm). (**F**) INS-1 cell lysates were immunoblotted with an antibody to phospho-PKR-like ER kinase (p-PERK) to measure ER stress in untreated control cells versus Tg-treated cells. Cells grown under standard culture conditions did not exhibit PERK phosphorylation as indicated by a higher, slower-migrating PERK-immunoreactive band (p-PERK). In contrast, 1 h and 6 h Tg-treated cells demonstrated p-PERK, indicating ER stress. (**G**) INS-1 cell lysates were also immunoblotted for additional ER stress and pro-apoptotic markers: phospho-eIF2 (p-eIF2α), a target of the PERK kinase, and the ER stress-induced pro-apoptotic transcription factor CHOP. Control cells exhibited no evidence of either p-eIF2 or CHOP expression. In contrast, 1 h Tg treatment induced p-eIF2α expression; longer 6h Tg treatment induced expression of both p-eIF2α and CHOP. 16 h Tm treatment induced CHOP expression but not eIF2α phosphorylation. There was no change in actin expression across all conditions. (**H**) Analysis of IRE1 activation by monitoring the levels of unspliced XBP1 (uXBP1) and spliced XBP1 (sXBP1) mRNA via RT-PCR. Standard culture conditions did not elicit XBP1 splicing in the controls whereas sXBP1 was detected in the 1 h Tg-treated condition.

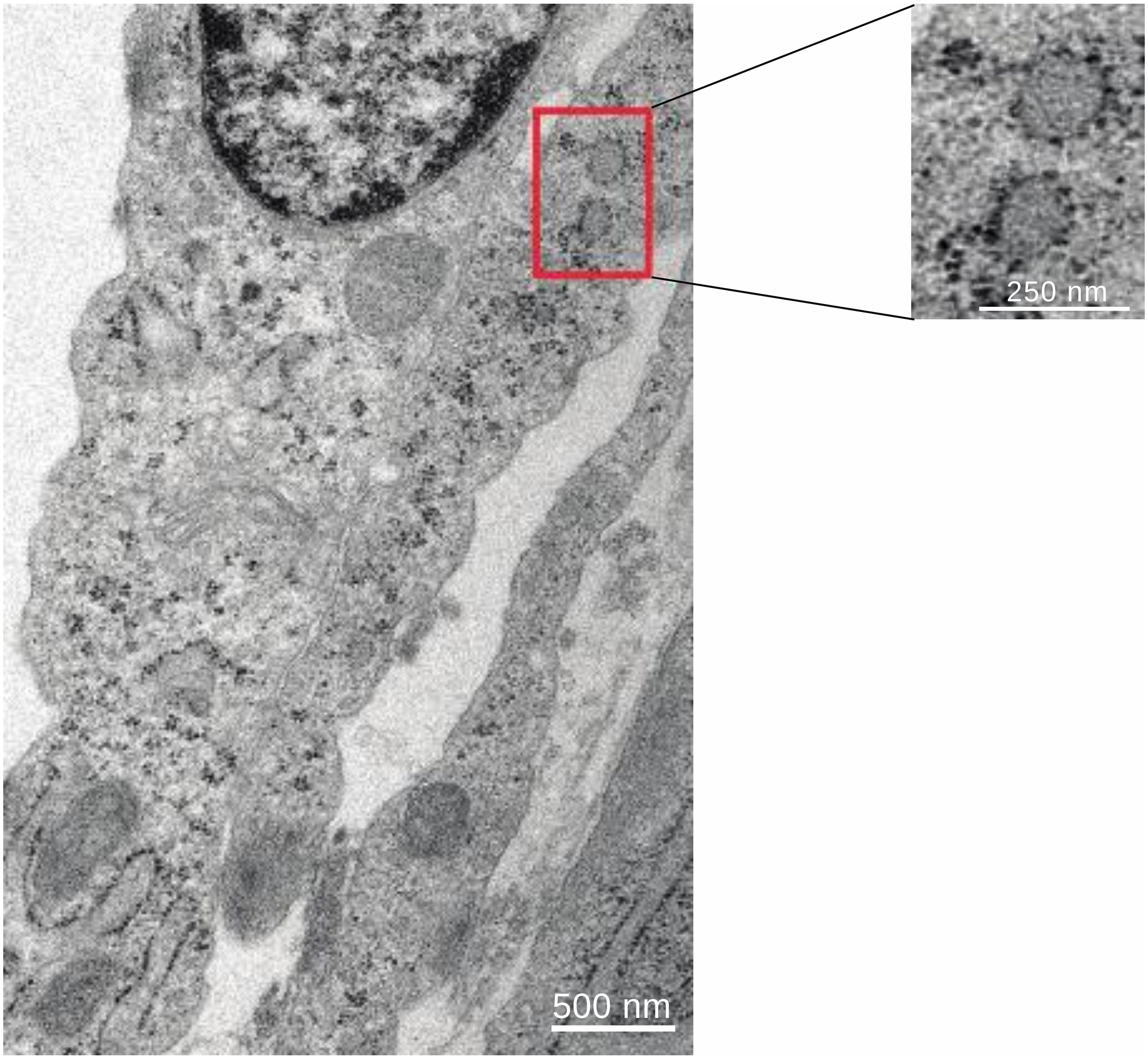

Fig. S12

Fig. S12. TEM evidence of RAVs in WI-38 cells. Two-dimensional evidence of ribosome-associated vesicular structures consistent with RAVs by conventional TEM in a peripheral cell extension of an untreated, untransfected WI-38 human lung fibroblast cell running along a neighboring cell; scale bar = 500 nm. An enlarged view of the RAVs from the highlighted region (red box) is alongside; scale bar = 250 nm.

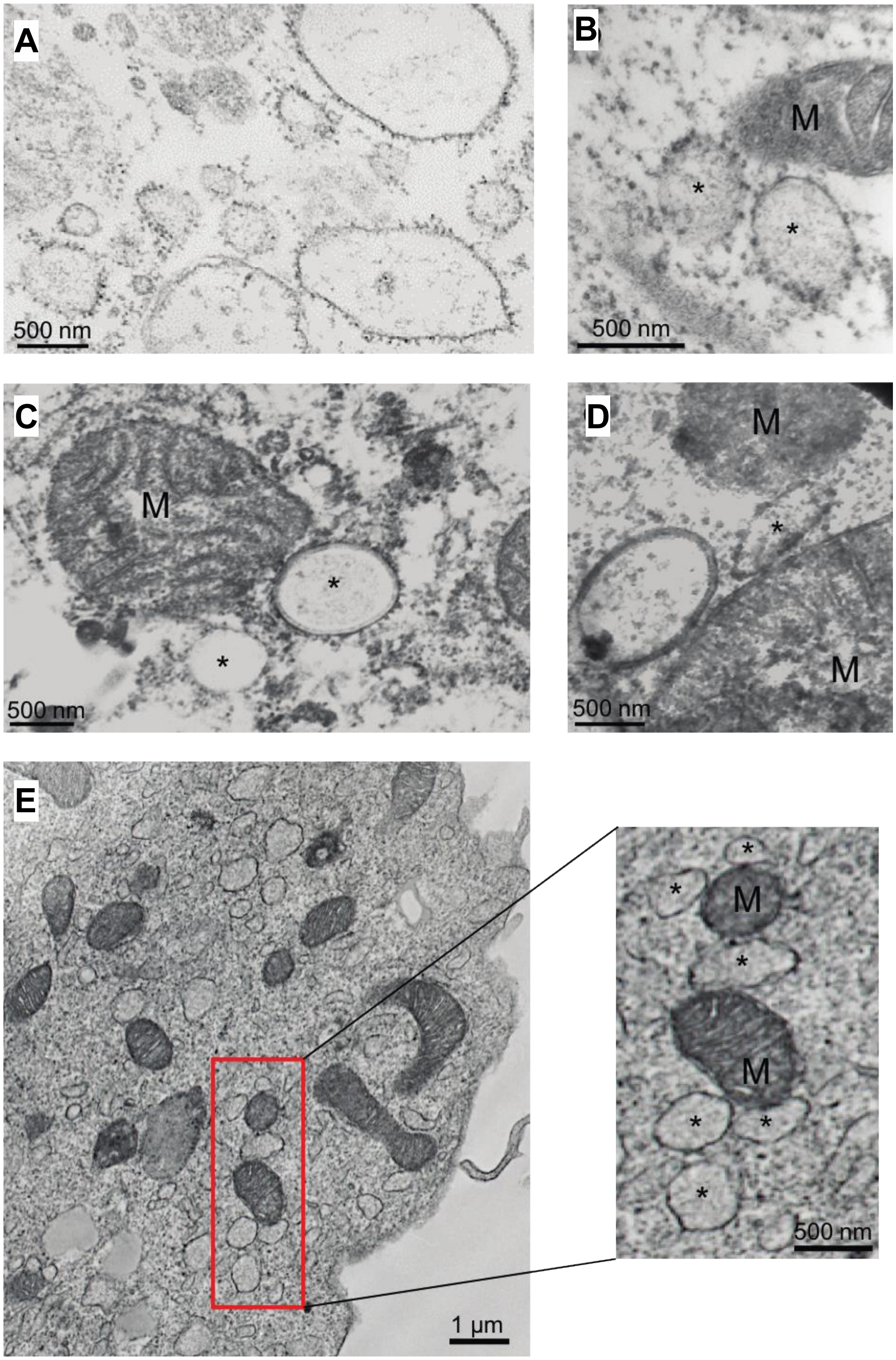

Fig. S13

Fig. S13. TEM evidence of RAVs in SW-13 cells. (**A-D**) Examples of RAVs in conventional two-dimensional TEM images of untreated, untransfected SW-13 cells, a human adrenocortical cell line; all scale bars = 500 nm. (**E**) Image of a peripheral region within a cell featuring RAVs associating with mitochondria; scale bar = 1 μm. Alongside is an enlarged image of the region highlighted within the red box; scale bar = 500 nm. For all images, RAVs are indicated by asterisks and mitochondria by "M."

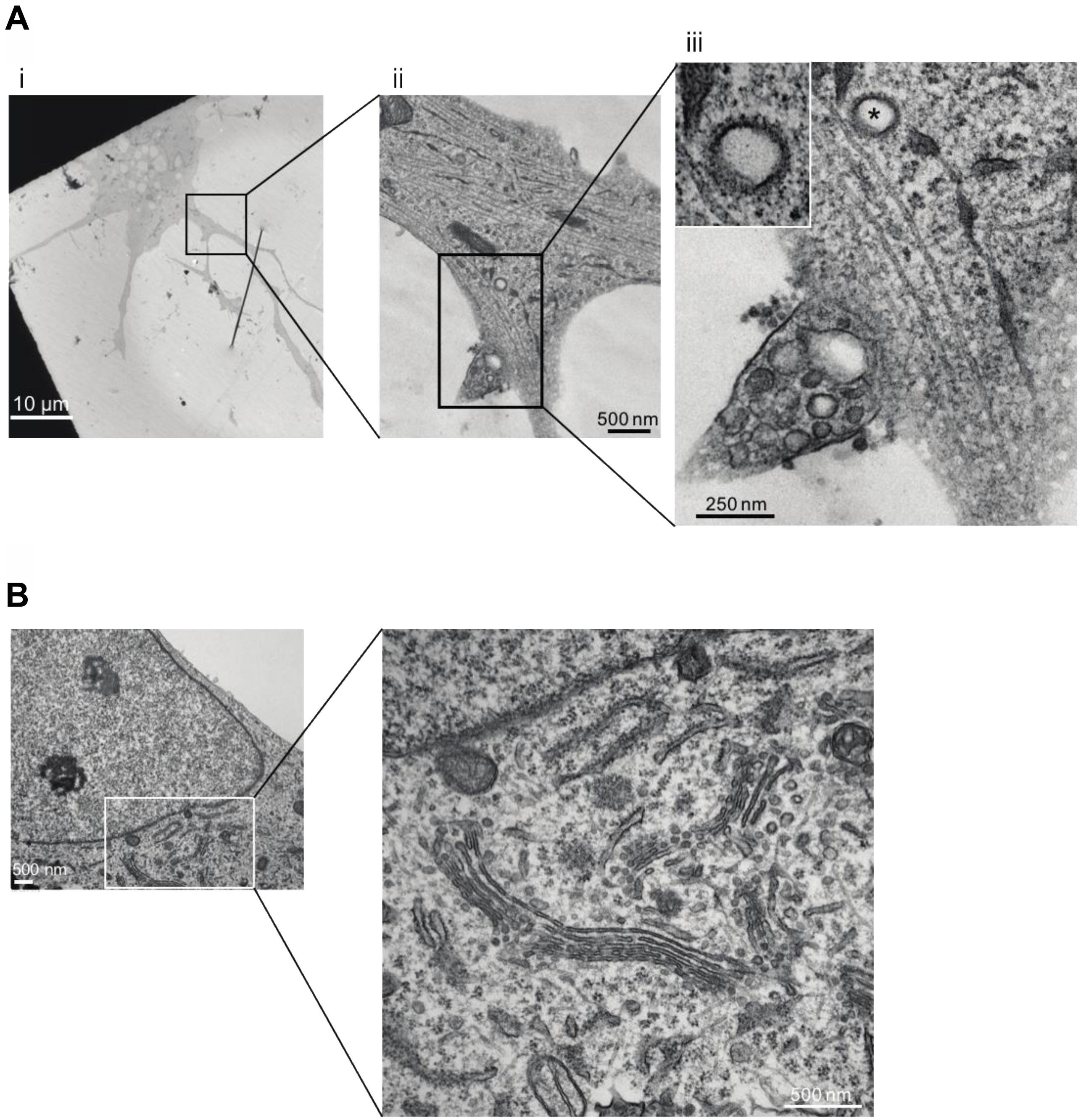

Fig. S14

Fig. S14. RAVs in developing primary hippocampal neurons. (**A**) Conventional TEM imaging of the periphery of a developing primary rat hippocampal neuron, featuring an extending dendrite (black box, **Panel i**; scale bar = 10 µm). Magnified view of a dendritic

protrusion (black box, **Panel ii**; scale bar = 500 nm). Further magnification of this dendritic region revealed a RAV (**Panel iii**, labeled by an asterisk; scale bar = 250 nm) in proximity to the membrane protrusion as well as vesicular structures and ribosomes within the protrusion itself; inset features a zoomed-in view of the RAV. (**B**) TEM micrograph of the central region of the neuron; scale bar = 500 nm. A magnified view of the perinuclear region (white box) demonstrated an intact, extensive secretory machinery including rough ER and numerous Golgi stacks; scale bar = 500 nm.

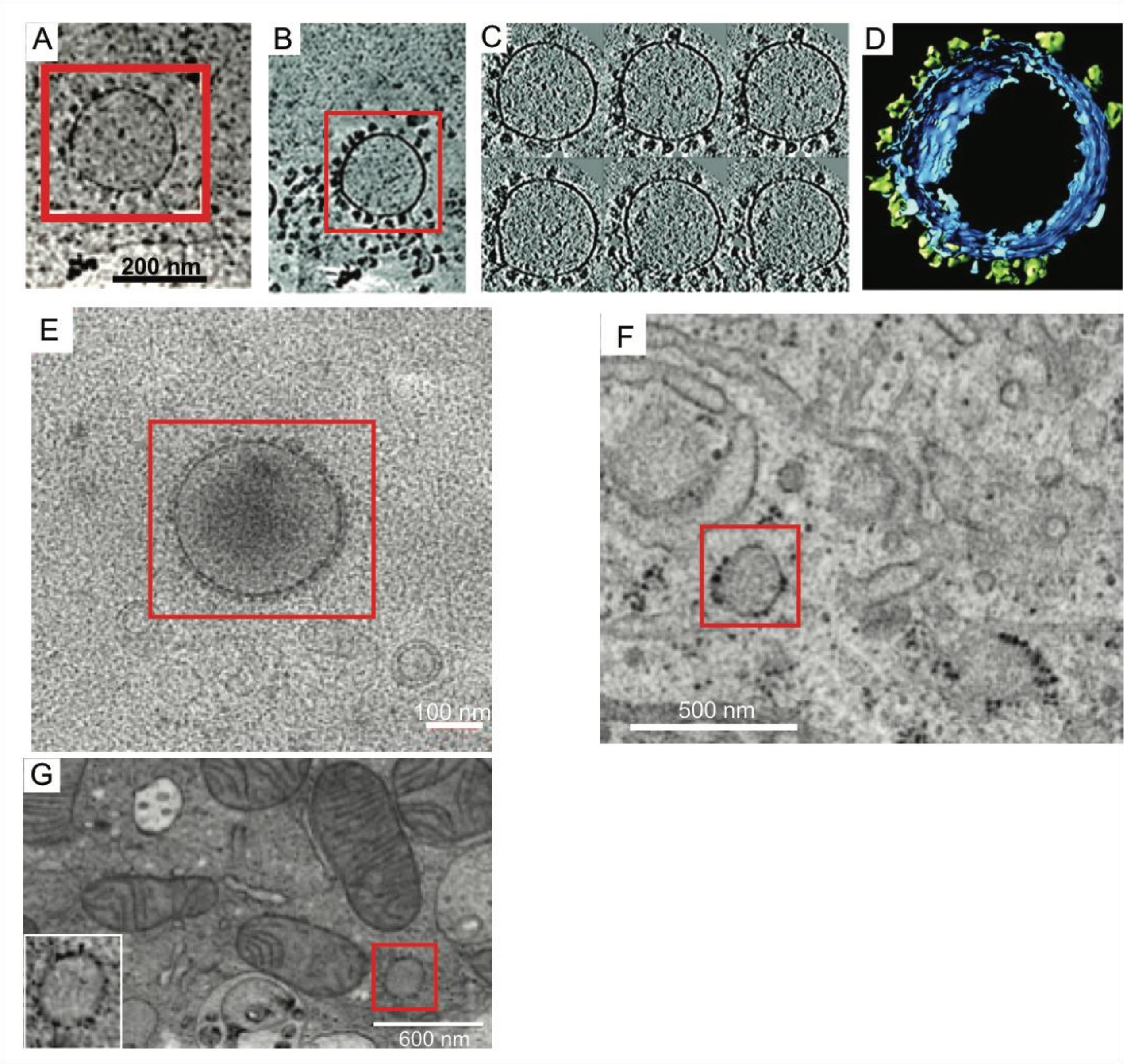

Fig. S15

Fig. S15. Evidence of RAVs in the literature. (**A**) Cryo-EM projection image of a peripheral region of a *Dictyostelium* cell highlighting a ribosome-associated vesicle consistent with RAV morphology and size (in red box); scale bar = 200 nm. The same peripheral region in *Dictyostelium* was used for cryo-ET imaging (**Panels B-D**). (**B**) A presumptive RAV (red box) was also observed in a tomogram x-y slice acquired from a tilt-series of vitrified *Dictyostelium*

cells. (**C**) Successive x-y slices through a tomogram of a RAV in *Dictyostelium*. (**D**) Rendered surface of the RAV structure in **Panel D**; all images from **Panels A-D** adapted from Medalia et al. Science 298, 1209-1213 (2002)(*52*). (**E**) x-y slice taken from cryo-ET imaging in mouse embryonic fibroblasts grown on EM grids demonstrating a RAV; scale bar = 100 nm. Adapted from Faas et al. J Cell Biology 198, 457-469 (2012)(*90*). (**F**) TEM image of a RAV (red box) in high-pressure frozen, freeze-substituted human HepG2 human hepatoblastoma cells; scale bar = 200 nm. Adapted from Hess et al. J Structural Biology 130, 63-72 (2000)(*91*). (**G**) Conventional TEM image of a RAV (red box) within high-pressure frozen, freeze-substituted African green monkey Vero kidney epithelial cells; scale bar = 600 nm. Inset shows a higher magnification image of the RAV. Adapted from Hawes et al. J Microscopy 226, 182-189 (2007)(*92*).
